# *HSD3B1* links ileal steroid metabolism to bile acid regulation in patients with prostate cancer

**DOI:** 10.1172/JCI202725

**Published:** 2026-06-15

**Authors:** Nikou Fotouhi, Robert Diaz, Mohammad Alyamani, Yoon-Mi Chung, Gail West, Pranab K. Mukherjee, Alireza Abdshah, Robert A. Burgess, Samreen Jatana, Rana R. McKay, Florian Rieder, Mary-Ellen Taplin, Nima Sharifi

**Affiliations:** 1 Desai Sethi Urology Institute and; 2 Sylvester Comprehensive Cancer Center, University of Miami Miller School of Medicine, Miami, Florida, USA.; 3 Department of Inflammation and Immunity, Cleveland Clinic Research, and; 4 Department of Gastroenterology, Hepatology & Nutrition, Digestive Diseases Institute, Cleveland Clinic Foundation, Cleveland, Ohio, USA.; 5 University of California San Diego, San Diego, California, USA.; 6 Department of Medical Oncology, Dana-Farber Cancer Institute, Boston, Massachusetts, USA.

**Keywords:** Endocrinology, Metabolism, Oncology, Prostate cancer

## Abstract

Androgen deprivation therapy (ADT), a cornerstone of advanced prostate cancer treatment, effectively suppresses androgen signaling but frequently induces systemic metabolic dysregulation. Here, we delineate an unrecognized intestinal steroid/bile acid regulatory axis that mechanistically links androgen suppression to extratumoral metabolic aberrations. *HSD3B1* is the most common inherited link to prostate cancer mortality and mediates its effects by regulating steroid metabolism. Integrated metabolomic profiling of patients undergoing ADT revealed a rapid genotype-associated reduction in circulating bile acids, most pronounced in carriers of the adrenal-permissive *HSD3B1* (1245C) allele. Surprisingly, analyses in human intestinal tissue and mechanistic investigations in in vitro models identified the terminal ileum as a unique site of *HSD3B1* and *SLC10A2* (ASBT) coexpression, where catalytically active 3βHSD1 is transcriptionally governed by liver receptor homolog-1 (LRH-1). Pharmacologic or genetic LRH-1 inhibition coordinately suppressed *HSD3B1* and *SLC10A2* expression and function, while inducing adaptive *HSD11B2* upregulation and enhanced glucocorticoid inactivation. This LRH-1–dependent regulatory program persisted independently of androgen and glucocorticoid receptor signaling under in vitro conditions modeling androgen deprivation. These findings establish LRH-1 as a central integrator of intestinal steroidogenesis and bile acid transport and implicate the LRH-1/*HSD3B1*/*SLC10A2* network as a mechanistic driver of ADT-associated metabolic disturbances and a potential target for therapeutic intervention.

## Abstract 2

This story unveils a surprising link between HSD3B1, a gene linked to prostate cancer mortality, with bile acid regulation in prostate cancer.

## Introduction

Prostate cancer is among the most prevalent malignancies and a leading cause of cancer-related death in men worldwide. Despite advances in detection and therapy, metastatic and high-risk disease remain associated with substantial morbidity and mortality (1, 2). Androgen deprivation therapy (ADT), achieved surgically or pharmacologically, is the cornerstone of treatment for locally advanced or metastatic prostate cancer (3, 4), often combined with next-generation androgen receptor (AR) pathway inhibitors such as enzalutamide, apalutamide, abiraterone, or darolutamide (5–7). By lowering circulating gonadal testosterone to castrate levels, ADT effectively suppresses androgen-dependent tumor growth. However, sustained androgen suppression induces widespread metabolic aberrations that contribute to therapeutic resistance, disease progression, and systemic toxicities including metabolic, cardiovascular, musculoskeletal, and neurocognitive complications, profoundly affecting survival and quality of life (3, 8, 9). These adverse effects underscore the dual challenge of maintaining oncologic control while mitigating the systemic metabolic consequences of androgen depletion in prostate cancer. Yet, the molecular mechanisms linking androgen suppression to extratumoral metabolic dysfunction, and their clinical implications, remain poorly understood, limiting our ability to predict or intervene against therapy-related toxicity and resistance.

The enzyme 3β-hydroxysteroid dehydrogenase type 1 (3βHSD1; encoded by *HSD3B1*) catalyzes an irreversible, rate-limiting conversion of adrenal dehydroepiandrosterone (DHEA) to androstenedione (AD), controlling the local tissue availability of potent androgens such as testosterone and dihydrotestosterone (10). We previously showed that in prostate cancer cells, a common germline variant in *HSD3B1* (the adrenal-permissive 1245C allele) encodes a gain-of-function enzyme that enhances intracrine androgen synthesis from adrenal-derived precursors and promotes resistance to ADT compared with the adrenal-restrictive 1245A allele. This *HSD3B1* variation is the most common inherited single-gene link to prostate cancer progression and mortality, enabling resistance to ADT and thereby reducing overall survival (10–14). However, the role of *HSD3B1* beyond the tumor microenvironment and its impact on systemic steroid metabolism during ADT remain poorly understood. In particular, the contribution of intestinal 3βHSD1 activity to systemic metabolism under ADT has never been explored. Recent metabolomic studies reveal alterations in circulating bile acid pools in prostate cancer patients receiving ADT (15–18), suggesting a previously unrecognized intersection between steroidogenesis and bile acid metabolism in extratumoral tissues. Because bile acids act as endocrine regulators of lipid, glucose, and energy metabolism in peripheral tissues (19), such changes may contribute to ADT-related metabolic complications. Systemic bile acid homeostasis depends on an extensive coordinated crosstalk between the liver and intestine. The small intestine, particularly the terminal ileum, absorbs approximately 95% of intestinal bile acids and plays a central role in enterohepatic bile acid circulation (20–22). This process is driven by active bile acid reuptake through the apical sodium-dependent bile acid transporter (SLC10A2, also known as ASBT), which also modulates host–microbiome interactions and metabolic signaling (23, 24).

We hypothesized that extraprostatic *HSD3B1* expression contributes to systemic steroid and bile acid reprogramming during ADT. Because the liver is canonically considered the primary site of bile acid regulation (19), we first explored hepatic tissue and then investigated the intestine as a candidate site of *HSD3B1*-mediated steroid–bile acid crosstalk. LRH-1 is a major nuclear receptor regulator of metabolism in the liver, intestines, and pancreas (25). Given its established role in intestinal epithelial homeostasis, we prioritized LRH-1 as a candidate upstream regulator of *HSD3B1* and *SLC10A2*. Here, we identified a distinct ileal 3βHSD1-mediated mechanism in intestinal enterocytes that functions independently of canonical steroid receptors yet is tightly regulated by LRH-1. We showed that LRH-1 coordinately regulates *HSD3B1* and *SLC10A2*, thereby linking intestinal steroidogenesis with bile acid homeostasis. Notably, LRH-1 inhibition induces adaptive expression of 11β-hydroxysteroid dehydrogenase type 2 (*HSD11B2*), enhancing glucocorticoid inactivation and further decoupling this pathway from canonical steroid receptors. Collectively, our findings reveal an unrecognized intestine-specific steroidogenic adaptation that associates *HSD3B1* genotype to systemic bile acid remodeling and glucocorticoid homeostasis during androgen deprivation. These insights suggest that targeting LRH-1– or 3βHSD1-dependent signaling may mitigate ADT-associated metabolic toxicity and therapeutic resistance. By redefining the intestine as an active participant in the host metabolic response to ADT, this work establishes a mechanistic link between germline *HSD3B1* inheritance, bile acid metabolism, and systemic host response to androgen deprivation in prostate cancer.

## Results

### Androgen deprivation and apalutamide therapy induce reductions in circulating bile acids.

**Figure 1:**
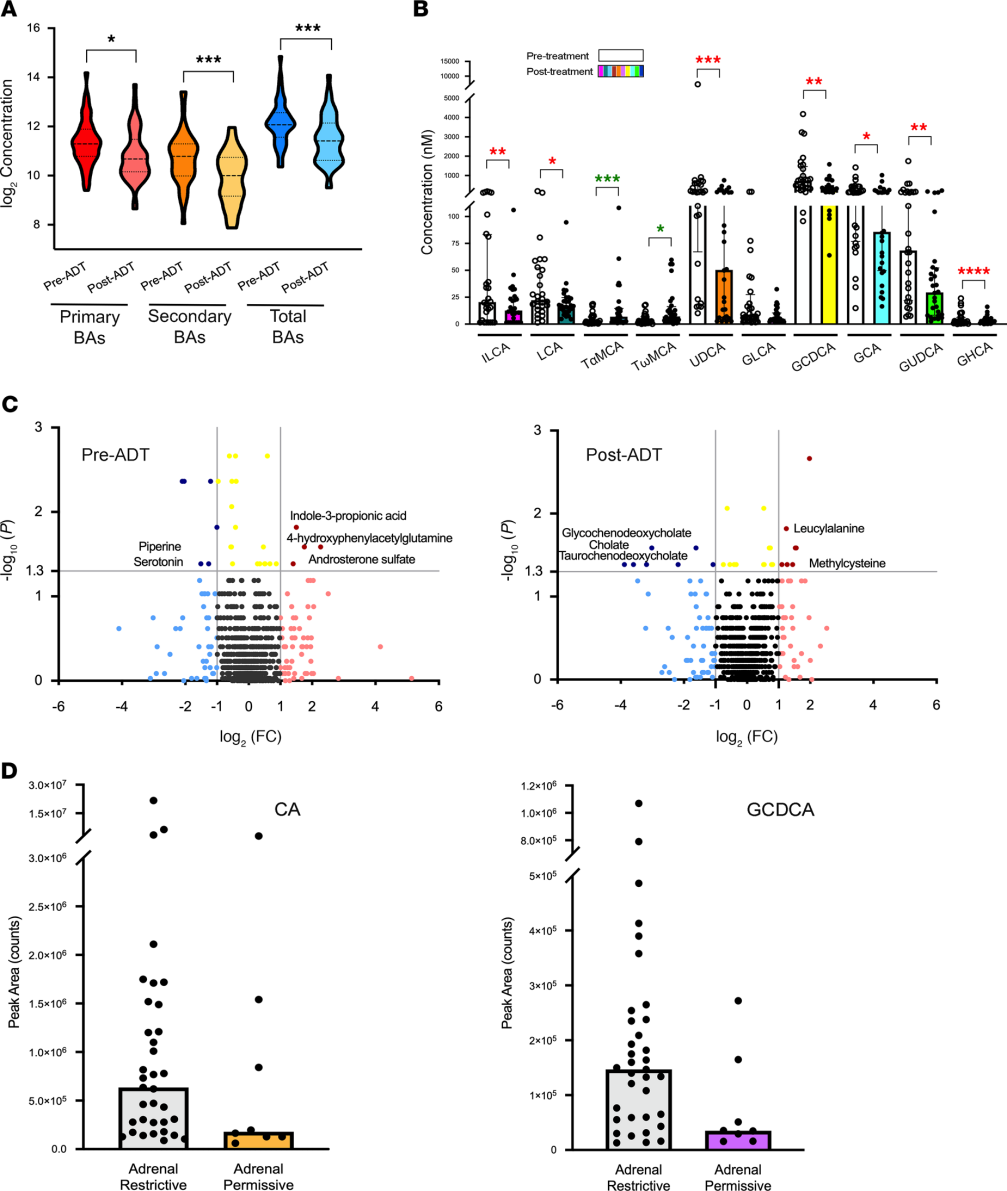
Metabolic profiling of circulating bile acids in patients with prostate cancer receiving ADT plus androgen pathway inhibitor. (**A** and **B**) Targeted metabolomic analysis of circulating bile acids (BAs) in 46 patients before and 28 days after treatment demonstrated significant reductions in primary, secondary, and total bile acids, independent of *HSD3B1* genotype. (**C**) Untargeted metabolomic analysis with genotype stratification revealed greater posttreatment declines in homozygous adrenal-permissive (*n* = 6) compared with homozygous adrenal-restrictive (*n* = 6) patients, particularly for TCDCA, GCDCA, and CA. Dark blue dots in the graphs represent metabolites that are significantly higher in adrenal-restrictive HSD3B1 genotype, and red dots represent metabolites that are significantly higher in adrenal-permissive HSD3B1 genotype. (**D**) Targeted metabolomics of an independent cohort (Dana-Farber Cancer Institute) including adrenal-restrictive (*n* = 35) and adrenal-permissive (*n* = 8) patients after 6 months of ADT confirmed sustained reductions in CA and GCDCA. Data are presented as median ± SD; Mann-Whitney *U* test; *P < 0.05, **P < 0.01, ***P < 0.001, ****P < 0.0001.

To investigate the systemic metabolic consequences of hormonal therapy, we conducted comprehensive untargeted serum metabolomic profiling in prostate cancer patients receiving ADT combined with the AR antagonist apalutamide in the neoadjuvant setting prior to prostatectomy. Unbiased metabolomic analysis revealed extensive therapy-associated metabolic reconfiguration, with striking alterations observed in circulating bile acids (Figure 1). Our targeted metabolomic analyses showed that circulating levels of primary, secondary, and total bile acids declined significantly 28 days after treatment initiation (Figure 1, A and B, and Supplemental Table 1; supplemental material available online with this article; https://doi.org/10.1172/JCI202725DS1). With stratification by *HSD3B1* genotype, bile acid profiles exhibited distinct genotype-associated differences, consistent with the enzyme’s role in extragonadal biosynthesis. Notably, patients with homozygous adrenal-permissive inheritance showed a greater decline in primary bile acids, including taurochenodeoxycholic acid (TCDCA), glycochenodeoxycholic acid (GCDCA) and cholic acid (CA), compared with those with homozygous adrenal-restrictive inheritance, as shown in the untargeted analysis (Figure 1C). Across the cohort, median absolute posttreatment changes revealed a 4.6-fold greater reduction in TCDCA, a 1.9-fold greater reduction in GCDCA, and a 2.7-fold greater reduction in cholic acid in adrenal-permissive individuals relative to adrenal-restrictive carriers. To validate these findings, targeted metabolic profiling was performed on an independent cohort of patients receiving ADT plus AR pathway inhibitor for 6 months (26, 27). Consistent with the initial cohort, the second cohort also showed pronounced reduction in circulating primary bile acids, including GCDCA and CA, following therapy, with a more pronounced decline observed in adrenal-permissive individuals (Figure 1D). Together, these data demonstrate that androgen deprivation plus AR inhibitor is associated with reproducible, genotype-dependent reductions in circulating bile acids, highlighting a clinically relevant metabolic alteration associated with systemic androgen suppression.

### HSD3B1 and SLC10A2 are exclusively coexpressed and functionally active in human ileal enterocytes.

**Figure 2:**
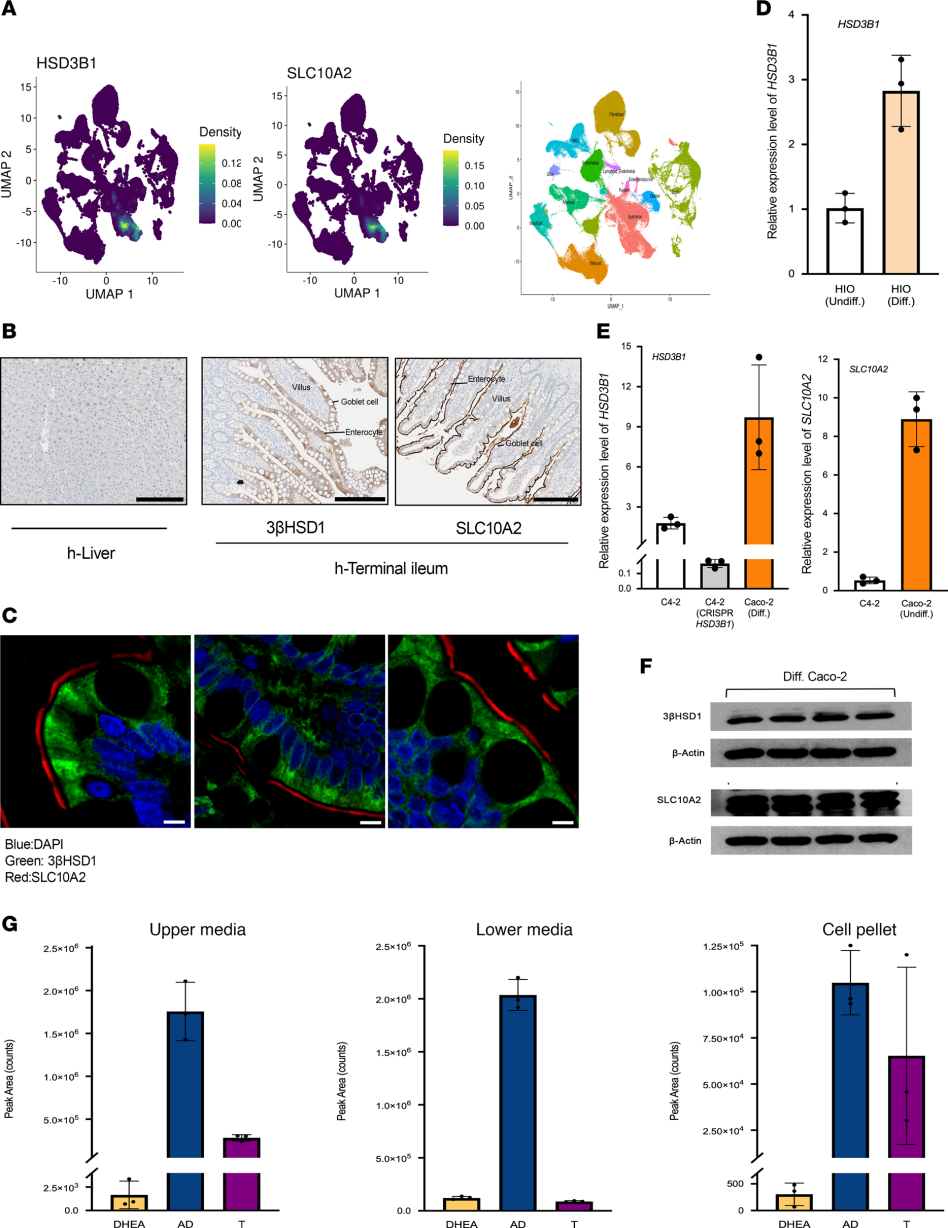
3βHSD1 and SLC10A2 are exclusively coexpressed and functionally active in human ileal enterocytes. (**A**) scRNA-seq of human gastrointestinal tissues showed selective *HSD3B1* enrichment in terminal ileal epithelial cells, overlapping with *SLC10A2* (ASBT). (**B** and **C**) IHC and immunofluorescence of human liver and terminal ileum (*n* = 4 independent tissue samples per tissue type) revealed exclusive colocalization of 3βHSD1 and SLC10A2 within enterocytes. Scale bars: 200 μm (**B**), 5 μm (**C**). In **C**, DAPI (blue), 3βHSD1 (green), and SLC10A2 (red). (**D**) RT-qPCR confirmed *HSD3B1* expression in undifferentiated (Undiff.) and differentiated (Diff.) HIOs. (**E** and **F**) qRT-PCR and Western blot analyses showed robust coexpression of *HSD3B1* and *SLC10A2* in Caco-2 cells, with moderate *HSD3B1* expression in C4-2 cells. (**G**) 3βHSD1 metabolic activity in differentiated Caco-2 cells assessed by 24 h DHEA (100 nM) treatment, yielding AD and testosterone (T), indicating enzymatic activity. Data are presented as mean ± SD; *n* = 3 independent experiments.

Given the pronounced decline in circulating bile acids in adrenal-permissive prostate cancer patients after ADT plus AR antagonist, we hypothesized that *HSD3B1* may contribute to bile acid metabolism or physiology independently of its role in prostate cancer. We suspected that there were 2 mechanisms by which bile acids could be regulated by *HSD3B1*: regulation of bile acid biosynthesis, which occurs in the liver, or enterohepatic bile acid cycling, which requires intestinal transport. To define the tissue-specific distribution of *HSD3B1* expression, we performed an integrated transcriptomic, proteomic, and functional analyses across human gastrointestinal and hepatic tissues. Despite the liver’s canonical role in bile acid biosynthesis, *HSD3B1* transcript and protein were undetectable in human liver, hepatocytes, or hepatic carcinoma cell lines (HepG2 and HuH-7) (Supplemental Figure 1, A–C). This absence effectively excludes a role for *HSD3B1* in hepatic bile acid biosynthesis. To explore potential compensatory enzymes in hepatocytes, we combined sequence homology, conserved domain alignment, and liver transcriptomic analyses across the *HSD3B* family. Among these, *HSD3B7* displayed the highest sequence similarity and closest phylogenetic proximity to *HSD3B1* (28), suggesting possible functional overlap. HEK293T cells stably expressing *HSD3B7* converted 7α-hydroxycholesterol (C5) to 7α-hydroxy-4-cholestene-3-one (C4), confirming 3βHSD7 activity in a substrate- and time-dependent manner (Supplemental Figure 2A). In contrast, *HSD3B1* knockdown in C4-2 cells did not affect C4 production from C5, indicating that 3βHSD1 cannot substitute for the biochemical function of 3βHSD7 (Supplemental Figure 2B). Moreover, unlike 3βHSD1, *HSD3B7*-expressing HEK293T cells failed to convert DHEA to AD or testosterone, demonstrating 3βHSD7 lacks steroidogenic activity (Supplemental Figure 2C). Given the absence of hepatic *HSD3B1* expression and the lack of compensatory steroidogenic activity by *HSD3B7*, making it unlikely that *HSD3B1* contributes to bile acid biosynthesis, we next explored the second possible mechanism of enterohepatic circulation by examining extrahepatic sites of *HSD3B1* expression, focusing on the small intestine, as a potential site of steroidogenic–bile acid crosstalk. Strikingly, scRNA-seq of human intestinal tissues revealed marked enrichment of *HSD3B1* in terminal ileal epithelial cells, where it was tightly coexpressed with the apical sodium-dependent bile acid transporter *SLC10A2* (*ASBT*) (Figure 2A). This coexpression signature was absent in hepatocytes or colonic epithelium, designating the terminal ileum as a unique site of 3βHSD1 expression and potential activity. Protein-level validation by IHC and immunofluorescence of human terminal ileum confirmed exclusive colocalization of 3βHSD1 and SLC10A2 in ileal enterocytes, with a distinct crypt-to-villus gradient (Figure 2, B and C, and Supplemental Figure 3, A and B). To assess whether 3βHSD1 is catalytically active in ileal enterocytes, we next employed epithelial enterocyte–relevant in vitro models, including Caco-2 cells and human ileum organoids (HIOs). RT-qPCR and Western blot analyses confirmed robust expression of *HSD3B1* and *SLC10A2* in both Caco-2 cells and ileal organoids (Figure 2, D–F). Notably, *HSD3B1* transcript abundance was substantially higher in Caco-2 cells than in C4-2 prostate cancer cells, consistent with an intestinal physiological role. To complement these molecular observations, functional steroid conversion assays confirmed enzymatic activity of 3βHSD1. Treatment of differentiated Caco-2 monolayers with 100 nM DHEA for 24 h resulted in a rapid production of AD and testosterone, the latter predominantly in the apical compartment (Figure 2G), confirming metabolically active 3βHSD1 in human enterocytes. These findings were also recapitulated in HIOs (Supplemental Figure 4). Importantly, the parallel demonstration of both expression and enzymatic function emphasizes that 3βHSD1 in the intestinal epithelium is not merely a marker of steroidogenic potential but an enzymatically active contributor to local steroid metabolism. Collectively, these results identify the terminal ileum as the exclusive site of *HSD3B1* and *SLC10A2* coexpression and establish 3βHSD1 as an enzymatically active enterocyte protein, alluding to a potential coordinating role in local steroid metabolism and bile acid uptake.

### LRH-1 drives the intestinal HSD3B1/SLC10A2 coregulation through an AR-independent mechanism.

**Figure 3:**
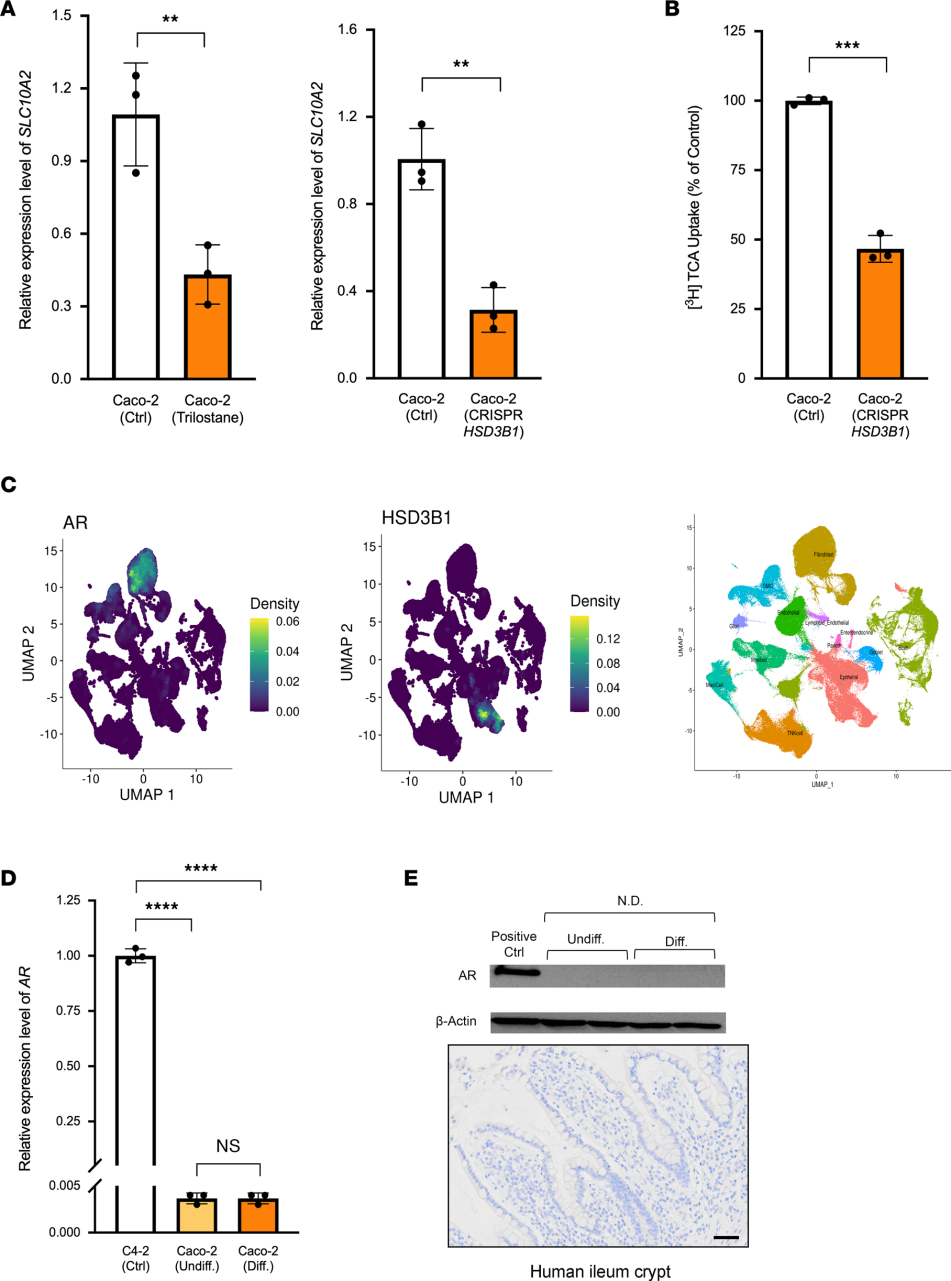
Bile acid transport in ileal enterocytes occurs independently of AR signaling. (**A**) Chemical inhibition of 3βHSD1 with trilostane (25 μM) or CRISPR/Cas9-disrupted *HSD3B1* each significantly reduced *SLC10A2* expression in Caco-2 cells. (**B**) Bile acid uptake assays showed decreased TCA transport in CRISPR/Cas9-disrupted *HSD3B1* Caco-2 cells. (**C**–**E**) AR was undetectable in human terminal ileum enterocytes. UMAP analysis of intestinal epithelial cells revealed no overlap between *AR* and *HSD3B1*, consistent with absence of AR transcript (RT-qPCR) and protein (Western blot) in Caco-2 cells and ileal crypts (*n* = 3 independent tissue samples). Scale bar: 100 μm. Undifferentiated (Undiff.) and differentiated (Diff.) Caco-2 cells are shown. Data are presented as mean ± SD; *n* = 3 independent experiments. Statistical significance was determined using a 2-tailed Student’s *t* test or, for comparisons of more than 2 groups with a common control, 1-way ANOVA followed by Dunnett’s multiple-comparison test; **P < 0.01, ***P < 0.001, ****P < 0.0001.

To further delineate regulatory mechanisms governing *HSD3B1* and *SLC10A2* expression in ileal enterocytes, we first assessed the impact of 3βHSD1 functional disruption using complementary pharmacologic and genetic approaches. Treatment with trilostane (25 μM) (29) or CRISPR-mediated *HSD3B1* disruption significantly decreased *SLC10A2* mRNA expression (Figure 3A) and attenuated taurocholic acid (TCA) transport in Caco-2 cells, suggesting that *HSD3B1* activity is required to maintain bile acid transport capacity in enterocyte models (Figure 3B). We next examined whether AR signaling contributes to *HSD3B1* and *SLC10A2* regulation in the ileum. Surprisingly, across complementary analytical modalities, no evidence of AR expression was detected in ileal epithelial cells. scRNA-seq (UMAP) analysis of human intestinal tissues revealed no overlap between *AR* and *HSD3B1* expression across epithelial populations (Figure 3C). Consistently, AR transcripts and protein were undetectable in Caco-2 cells and human ileal crypt epithelium by RT-qPCR and Western blot (Figure 3, D and E). These findings excluded AR involvement as a driver of *HSD3B1/SLC10A2* regulation in ileal enterocytes and prompted investigation of alternative transcriptional control mechanisms.

**Figure 4:**
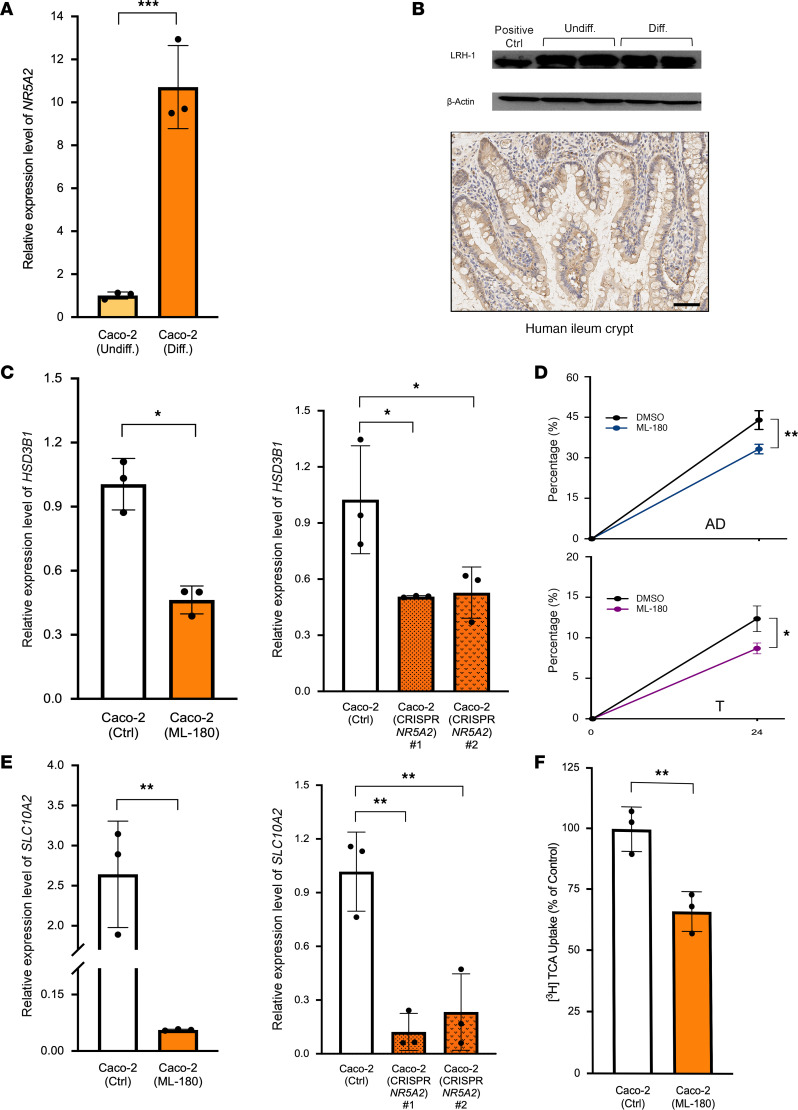
LRH-1 drives the *HSD3B1*/*SLC10A2* axis regulating bile acid transport. (**A** and **B**) *NR5A2* (LRH-1) was ubiquitously expressed in human ileal crypts and increased during Caco-2 differentiation (*n* = 4 independent tissue samples). Scale bar: 100 μm. (**C** and **D**) LRH-1 inhibition (ML-180, 15 μM) or CRISPR/Cas9 disruption of *NR5A2* decreased 3βHSD1 expression and reduced the conversion of DHEA to AD and testosterone (T) in differentiated Caco-2. (**E** and **F**) LRH-1 inhibition (ML-180, 10 μM) also significantly reduced *SLC10A2* expression and consequently decreased TCA transport. Undifferentiated (Undiff.) and differentiated (Diff.) Caco-2 cells are shown. Data are presented as mean ± SD; *n* = 3 independent experiments. Statistical significance was determined using a 2-tailed Student’s *t* test or, for comparisons of more than 2 groups with a common control, 1-way ANOVA followed by Dunnett’s multiple-comparison test; *P < 0.05, **P < 0.01, ***P < 0.001.

We therefore turned our focus to *NR5A2* (LRH-1), a nuclear receptor with established roles in maintaining intestinal epithelial integrity, homeostasis, and bile acid metabolism, although its function has been more extensively studied and characterized in the liver (25, 30, 31). Expression profiling by RT-qPCR and Western blot showed LRH-1 was robustly and ubiquitously expressed in Caco-2 cells and human ileal crypts (Figure 4, A and B). To test its functional contribution, we inhibited LRH-1 using the selective chemical antagonist ML-180 (10 μM) (32) or generated CRISPR-mediated *NR5A2*-knockdown alleles. Both approaches significantly reduced *HSD3B1* transcripts and 3βHSD1 activity, reflected by decreased DHEA-to-AD and -testosterone conversion (Figure 4, C and D). Parallel analyses revealed that LRH-1 inhibition significantly downregulated *SLC10A2* and impaired TCA uptake, suggesting coordinated transcriptional regulation (Figure 4, E and F). Collectively, these results identify LRH-1 as a key upstream regulator of the intestinal *HSD3B1/SLC10A2* axis. This LRH-1–dependent pathway operates independently of AR signaling, defining a distinct intestinal transcriptional program that coordinately regulates local steroid metabolism and bile acid transport.

### LRH-1 maintains intestinal HSD3B1/SLC10A2 regulation independently of glucocorticoid receptor signaling under ADT conditions.

**Figure 5:**
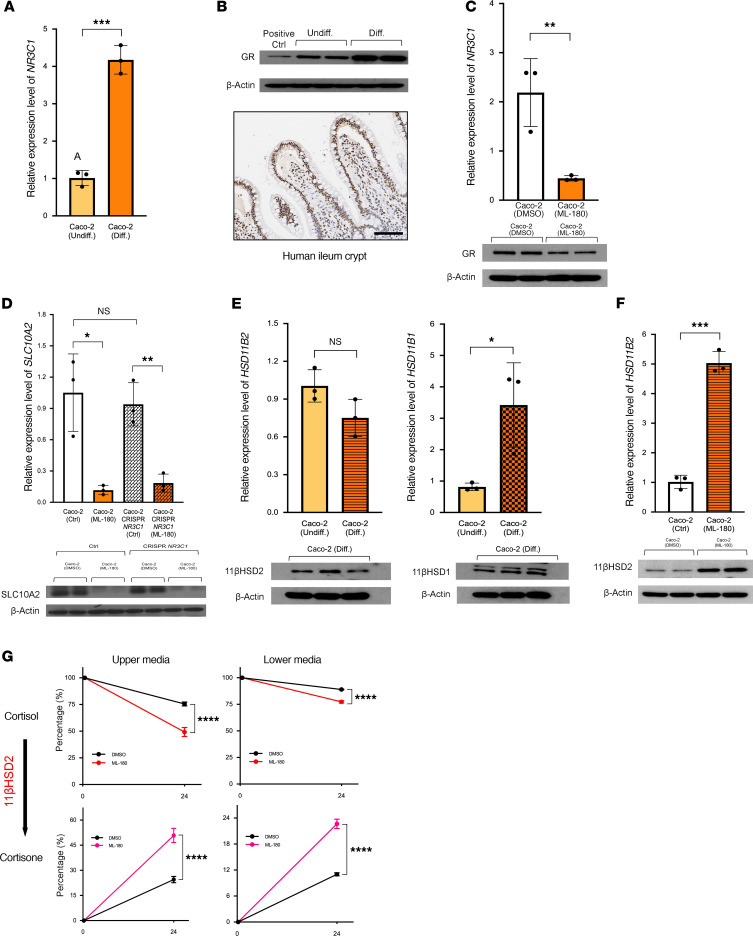
LRH-1 regulates the *HSD3B1/SLC10A2* axis independently of GR signaling under in vitro modeling of ADT. (**A** and **B**) RT-qPCR and Western blot analyses demonstrated GR expression in undifferentiated and differentiated Caco-2 cells. IHC of human terminal ileum (*n* = 4 independent tissue samples) confirmed nuclear GR localization in crypt epithelia. Scale bar: 100 μm. (**C**) LRH-1 inhibition (ML-180, 10 μM) significantly reduced GR transcript and markedly reduced protein levels, indicating LRH-1–dependent regulation. (**D**) Under charcoal-stripped serum conditions to model androgen- and glucocorticoid-depleted conditions, *SLC10A2* expression remained responsive to LRH-1 inhibition but was unaffected by GR disruption. (**E**) RT-qPCR and Western blot analyses demonstrated dynamic regulation of *HSD11B1* and *HSD11B2* during Caco-2 differentiation. (**F**) ML-180 treatment induced *HSD11B2* expression. (**G**) HPLC-based enzymatic assays using tritiated steroids demonstrated increased 11βHSD2 activity following LRH-1 inhibition, evidenced by enhanced cortisol-to-cortisone conversion in both apical (upper) and basolateral (lower) compartments at 0 and 24 h. Data are presented as mean ± SD; *n* = 3 independent experiments. Statistical significance was determined using a 2-tailed Student’s *t* test or, for comparisons of more than 2 groups with a common control, 1-way ANOVA followed by Dunnett’s multiple-comparison test; *P < 0.05, **P < 0.01, ***P < 0.001, ****P < 0.0001.

Having established LRH-1 as a principal regulator of the *HSD3B1/SLC10A2* axis, we next examined the contribution of glucocorticoid receptor (GR) signaling to modulation of this axis in ileal enterocytes, particularly under androgen deprivation conditions. We first profiled GR (*NR3C1*) expression across in vitro and in vivo models. GR transcripts and protein were detectable in both (un)differentiated Caco-2 cells by RT-qPCR and Western blot and localized predominantly in nuclei in human ileal epithelial crypts, consistent with active GR signaling in this compartment (Figure 5, A and B). We next asked whether LRH-1 regulates GR expression. Pharmacologic LRH-1 inhibition with ML-180 (10 μM) significantly reduced GR transcript and markedly reduced protein abundance, suggesting that GR is, at least in part, under the regulatory influence of LRH-1 (Figure 5C). Despite this regulatory link, we hypothesized that LRH-1 control of *SLC10A2* may persist independently of GR, particularly under androgen- and glucocorticoid-depleted conditions. To test this idea, we generated CRISPR-edited *NR3C1*-deficient Caco-2 cells and cultured them in charcoal-stripped serum medium. Strikingly, under this in vitro modeling condition, SLC10A2 transcript and protein levels remained responsive to LRH-1 perturbation yet were unaffected by GR loss, demonstrating LRH-1–mediated regulation of SLC10A2 independently of GR signaling (Figure 5D). Similar findings were observed following GR antagonist RU486 (mifepristone, 5 and 10 μM) treatment, supporting the notion that *SLC10A2* regulation may persist independently of GR activity (Supplemental Figure 5). Given that local glucocorticoid metabolism can strongly shape epithelial steroid signaling, we next profiled the expression of 11β-hydroxysteroid dehydrogenase type 1 (11βHSD1) and 11βHSD2. Both enzymes, especially 11βHSD1, were dynamically regulated during enterocyte differentiation, as shown by RT-qPCR and Western blot (Figure 5E). Intriguingly, LRH-1 inhibition with ML-180 significantly induced *HSD11B2* expression at both the mRNA and protein levels (~4-fold) (Figure 5F). Functional metabolic analysis confirmed that this induction was accompanied by a significant increase in 11βHSD2 activity, as reflected by enhanced cortisol-to-cortisone conversion (Figure 5G), suggesting a compensatory shift toward glucocorticoid inactivation. Taken together, these findings demonstrate that while LRH-1 positively controls GR expression, the intestinal *HSD3B1/SLC10A2* axis in ileal enterocytes remains independent of GR under in vitro conditions modeling androgen deprivation. LRH-1 inhibition further triggered compensatory *HSD11B2* upregulation, enhancing local glucocorticoid inactivation and further uncoupling SLC10A2 regulation from canonical GR signaling. This LRH-1–centered regulatory architecture suggests an adaptive intestinal steroidogenic reprogramming that may contribute to systemic metabolic alterations observed during ADT. We propose that LRH-1 functions as a tissue-intrinsic transcriptional regulator of intestinal steroidogenic and bile acid transport pathways, acting upstream of *HSD3B1*, *HSD11B2*, and *SLC10A2* and largely independently of GR signaling. In this model, LRH-1 induces *HSD3B1* expression, establishing a local steroidogenic program that supports bile acid handling and stabilizes *SLC10A2* expression. Importantly, LRH-1–dependent regulation of *HSD3B1* and *SLC10A2* can persist under conditions in which GR signaling is functionally constrained. In parallel, GR activity in intestinal epithelial cells is limited by LRH-1–controlled expression of 11βHSD2, which promotes glucocorticoid inactivation and restricts direct GR-dependent transcriptional control of *SLC10A2*. Thus, LRH-1 defines a dominant, GR-independent regulatory axis governing intestinal metabolic reprogramming.

### The LRH-1–driven intestinal steroidogenic circuit supports local metabolic adaptation during androgen deprivation.

**Figure 6:**
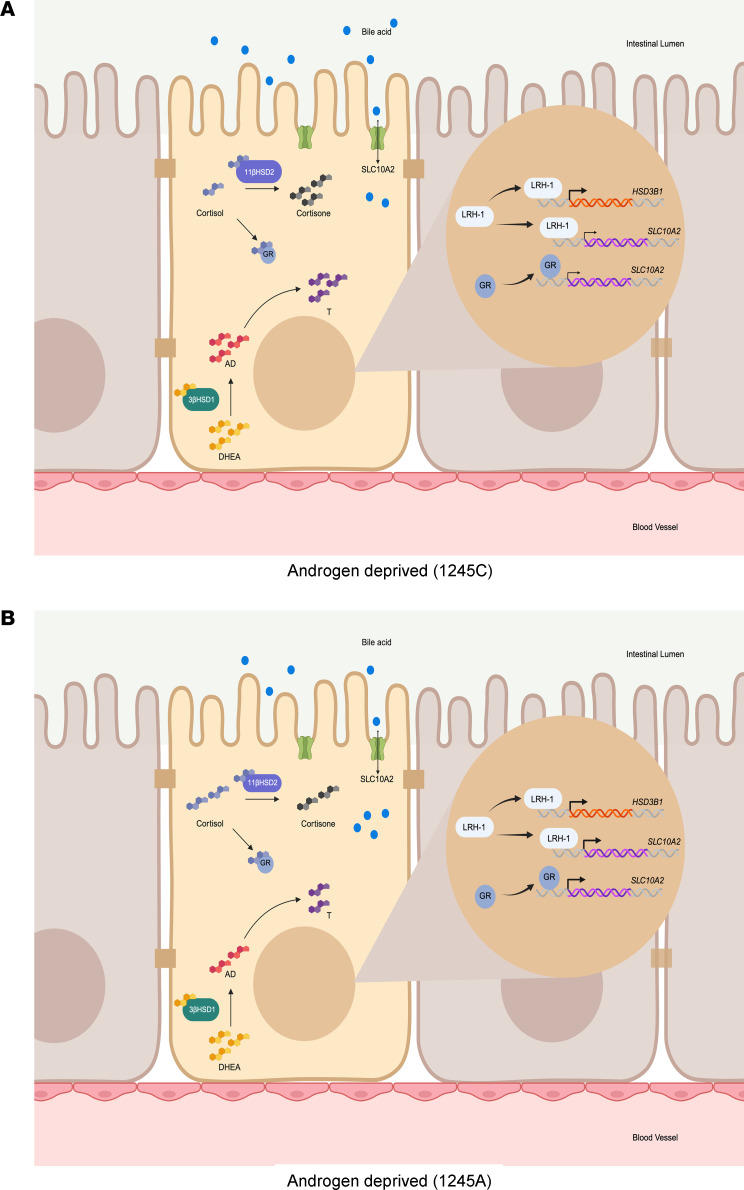
Integrated mechanistic model of intestinal bile acid regulation through the LRH-1/3βHSD1/SLC10A2 axis during ADT. (**A** and **B**) Schematic summarizing experimental findings. Under physiologic androgen-replete conditions, LRH-1 sustains *HSD3B1* and *SLC10A2* expression in terminal ileal enterocytes, supporting efficient bile acid uptake and recycling. LRH-1 also maintains local GR expression, and under androgen-replete conditions, GR signaling contributes to *SLC10A2* regulation. During androgen-deprived conditions, adrenal-permissive (*HSD3B1* 1245C) alleles encode a more stable 3βHSD1 enzyme that efficiently converts DHEA to downstream androgens, reducing dependence on LRH-1–driven *HSD3B1* transcription and thereby attenuating LRH-1 activity, including expression of downstream targets such as *SLC10A2*. In contrast, adrenal-restrictive (*HSD3B1* 1245A) alleles encode a less stable enzyme, maintaining reliance on LRH-1–mediated *HSD3B1* induction, which in turn modestly induces *SLC10A2* and other LRH-1 targets. In this context, LRH-1 remains the predominant regulator of the *HSD3B1/SLC10A2* axis, while GR signaling becomes functionally uncoupled from *SLC10A2* control. Reduced LRH-1 activity induces *HSD11B2* and local cortisol inactivation, further reinforcing the insulation of this pathway from canonical glucocorticoid signaling. Together, this model integrates genetic and therapeutic contexts to illustrate an intestinal steroid/bile acid loop during ADT. T, testosterone.

To synthesize our findings, we propose a working mechanistic model depicting how ADT rewires intestinal bile acid metabolism through an LRH-1/*HSD3B1/SLC10A2* regulatory axis (Figure 6, A and B). This model integrates patient genotype–metabolite associations with mechanistic observations derived from human intestinal tissue and epithelial model systems. Under androgen-replete conditions, LRH-1 maintains basal *HSD3B1* and *SLC10A2* expression in terminal ileal enterocytes, supporting coordinated local steroidogenesis and bile acid uptake in concert with GR. During ADT, germline *HSD3B1* adrenal-permissive (1245C) alleles encode a more stable 3βHSD1 variant that efficiently converts adrenal precursors (DHEA) into downstream androgens (AD and testosterone) without requiring increased *HSD3B1* transcription. In this context, LRH-1 need not be transcriptionally upregulated to maintain 3βHSD1 activity. LRH-1 also directly regulates *SLC10A2* and remains responsive to bile acid/FXR signaling; thus, its net activity and downstream bile acid transport may vary depending on intestinal cues and *HSD3B1* genotype. Thus, we propose that adrenal-permissive status may functionally decouple LRH-1–driven *HSD3B1* transcription from LRH-1–dependent bile acid transport control, resulting in genotype-associated differences in local bile acid handling. Importantly, we emphasize that this inference is based on mechanistic modeling rather than direct measurement of ileal bile acid flux in prostate cancer patients. Definitive proof of this model requires direct in vivo measurement of ileal LRH-1 activity, steroid conversion, and bile acid pools across *HSD3B1* genotypes under ADT conditions. Nevertheless, our integrated human and experimental data support the existence of an intestinal LRH-1/*HSD3B1/SLC10A2* circuit that influences local metabolic homeostasis of bile acids and steroids during androgen deprivation and provides a plausible mechanistic framework for genotype-associated gut-systemic metabolic adaptation observed clinically.

## Discussion

Androgen dependence is a defining hallmark of prostate cancer (33), and systemic androgen suppression through ADT leads to multisystemic metabolic complications that impair quality of life and increase cardiometabolic morbidity and other comorbidities in men with prostate cancer (3, 8, 34). However, the biological determinants underlying interpatient variability in ADT-associated adverse effects remain poorly defined. By suppressing gonadal androgens, ADT induces a profound hypogonadal state that perturbs whole-body metabolic homeostasis (35–37), a phenotype that has been independently associated with insulin resistance, adverse lipid profiles, increased adiposity, and features of metabolic syndrome in men receiving ADT and in hypogonadal states more broadly (38, 39). In addition, ADT shifts androgen predominance to circulating adrenocortical precursors (40), whereby 3βHSD1-mediated peripheral conversion of adrenal DHEA becomes a key determinant of residual androgenic signaling in peripheral tissues (41, 42). Clinical evidence across multiple prostate cancer cohorts demonstrates that the adrenal-permissive *HSD3B1* allele enhances 3βHSD1 stability and activity, accelerates androgen biosynthesis, and promotes cancer progression compared with the adrenal-restrictive allele (10, 12–14, 43–45). Here, we extend the role of *HSD3B1* beyond the tumor and identify it as a determinant of systemic metabolic shift during androgen deprivation. Emerging metabolomic data suggest that ADT perturbs systemic metabolism beyond classical lipid and glucose pathways, including bile acid homeostasis (16, 46). Perturbations in bile acid homeostasis are linked to altered lipid absorption, shifts in gut microbiome composition, and dysregulated hepatic-intestinal bile acid regulation (15, 47–50). In 2 independent cohorts, ADT plus direct AR blockade markedly lowered circulating bile acids, showing genotype-associated reductions in primary bile acids, with more pronounced depletion in adrenal-permissive *HSD3B1* (1245C) individuals. These findings support a genetic association between inherited steroidogenic activity and systemic metabolic responses, specifically bile acid metabolism, during ADT plus direct AR blockade. It is likely that similar broad metabolic changes may also occur with hormonal therapy that instead uses abiraterone for CYP17A1 blockade or enzalutamide monotherapy (51, 52). Mechanistic studies indicate that altered bile acid signaling through FXR and TGR5 can regulate glucose and lipid metabolism, hepatic steatosis, and energy expenditure. In mouse models, 6α-hydroxylated bile acids mediate TGR5 signaling and improve glucose homoeostasis in response to dietary fiber supplementation, providing direct evidence that specific bile acid species can modulate metabolic pathways (53–57). Consistent with these findings, multiple human studies demonstrated that alterations in bile acid abundance are strongly associated with metabolic phenotypes in other clinical contexts. Randomized phase III and IIb trials further show that bile acid–targeted interventions are associated with clinically meaningful modification of outcomes, including liver fibrosis, disease-associated biochemical markers, neoplastic risk reduction, and symptomatic benefit with systemic bile acid lowering (odevixibat) (58–61). Together, these observations support bile acid perturbations as a biologically relevant metabolic axis that may intersect with ADT-associated systemic metabolic effects, without establishing direct causality in the present cohorts. In this context, genotype-dependent bile acid depletion presents a plausible contributor to ADT-associated derangements such as dyslipidemia, insulin resistance, and hepatic steatosis.

To identify tissue-specific contributors to these systemic changes, we assessed *HSD3B1* expression and activity across the liver and small intestine. Integrated multiomic analyses revealed the terminal ileum as an underappreciated extragonadal niche of *HSD3B1* and peripheral steroidogenesis, positioning the intestine as a modulator rather than a passive recipient of hormonal perturbation during ADT. To our knowledge, we provide the first protein-level, cell-specific localization of 3βHSD1 in human terminal ileal enterocytes. Protein-level mapping demonstrated that 3βHSD1 colocalized with SLC10A2 in enterocytes. Mass spectrometry further confirmed active DHEA-to-androgen conversion in Caco-2 cells and ileal organoids, defining a specialized epithelial niche where steroidogenesis and bile acid transport converge. These results recast the intestine, traditionally viewed as a passive absorber, as an active steroid-metabolizing organ with the capacity to modulate systemic bile acid profiles and endocrine balance under ADT. Mechanistically, prior studies implicated glucocorticoids or GR overexpression in transcriptional activation of *SLC10A2* (62–64), while others suggested LRH-1 involvement (65, 66). Our data unify and extend this regulatory hierarchy by identifying LRH-1 (*NR5A2*) as the principle upstream transcriptional regulator of both *HSD3B1* and *SLC10A2* in human ileal epithelium. The *HSD3B1*-positive subpopulation of intestinal cells lacked AR but robustly expressed LRH-1, not SF-1, indicating a steroidogenic program independent of canonical AR-driven signaling. LRH-1 inhibition, either pharmacologic or genetic disruption of *NR5A2*, markedly suppressed *HSD3B1* and attenuated DHEA conversion, with concurrent reductions in *SLC10A2* transcription and TCA transport, supporting LRH-1 as a coordinator of intestinal steroidogenesis and bile acid homeostasis.

Under in vitro conditions modeling androgen deprivation, this LRH-1–dependent regulatory program persisted intact despite GR inactivation. Neither CRISPR-mediated GR knockdown nor RU486 treatment altered *SLC10A2* transcript or protein levels, suggesting that GR is dispensable for *HSD3B1*/*SLC10A2* regulation under androgen-deprived conditions. In contrast, LRH-1 inhibition induced *HSD11B2* expression and enhanced cortisol inactivation, supporting a compensatory mechanism that restricts epithelial glucocorticoid exposure while maintaining barrier integrity. This adaptive response may help preserve local glucocorticoid tone when systemic androgens are suppressed but, in doing so, may inadvertently perturb bile acid homeostasis and intestinal barrier signaling. Since LRH-1 is known to respond to phospholipids and bile acid–dependent proteins (30), and coregulates both *SLC10A2* and *HSD3B1*, our findings support the existence of a self-reinforcing intestinal metabolic circuit, in which LRH-1 activation enhances local steroidogenesis and bile acid transport.

Although Caco-2 cells and human intestinal organoids are well-validated and experimentally tractable models of enterocyte biology, they do not fully recapitulate the complexity of in vivo ileal physiology, including physiologic luminal bile acid gradients, enterohepatic dynamic cycling, microbiome-derived metabolites, and integrated enteroendocrine or neural signaling. Nevertheless, these models enable controlled interrogation of epithelial cell–intrinsic regulatory pathways, allowing isolation of LRH-1–dependent steroidogenic and bile acid transport programs independent of systemic confounders. Accordingly, our findings delineate epithelial cell–intrinsic mechanisms rather than reconstructing in vivo bile acid dynamics. Future in vivo studies including intestine-specific genetic perturbation of *NR5A2* or *HSD3B1* coupled with direct measurements of bile acid flux under androgen deprivation are required to validate and extend the physiological relevance of this model.

Together, these findings support that ADT induces an LRH-1–driven intestinal adaptive program, locally homeostatic yet systemically maladaptive, that sustains local steroidogenesis and modulates bile acid handling, independently of AR or GR signaling, despite systemic androgen suppression. Although this pathway may preserve epithelial homeostasis, sustained extra-adrenal steroid flux driven by *HSD3B1* upregulation (42), together with bile acid perturbation, may contribute to ADT-associated metabolic complications, including insulin resistance, hepatic steatosis, and increased cardiovascular risk. Notably, the magnitude of this response, bile acid reduction, was shaped by *HSD3B1* genotype; patients with adrenal-permissive alleles exhibited greater bile acid reduction, nominating it as a candidate biomarker for metabolic risk stratification during ADT.

Translationally, the LRH-1/3βHSD1/SLC10A2 axis represents a biologically tractable pathway for modulating intestinal steroid metabolism and bile acid reabsorption in the setting of ADT. Future studies should define whether selective intestinal 3βHSD1 inhibition or targeted LRH-1 modulation can restore bile acid reabsorption and mitigate maladaptive steroidogenesis without undermining systemic androgen suppression and should evaluate downstream consequences on lipid and glucose metabolism, enteroendocrine signaling, and microbiome-derived bile acid pools.

In summary, we define an LRH-1–dependent, GR-independent intestinal steroidogenic program that integrates bile acid homeostasis, inherited steroidogenic capacity, and metabolic health during androgen deprivation. By positioning LRH-1 upstream of *HSD3B1* and SLC10A2, we recast the intestine as a dynamic endocrine organ that responds to systemic hormonal stress. These findings provide a mechanistic framework for ADT-associated metabolic perturbation and identify LRH-1 as a potential therapeutic node to mitigate systemic complications while preserving oncologic efficacy.

## Methods

### Sex as a biological variable

Only male subjects were included, as this study focuses on prostate cancer, a male-specific disease. Therefore, sex was not considered as a biological variable. The findings are expected to be broadly relevant within the context of prostate cancer biology and treatment. 

### Clinical samples and bile acid quantification

#### Cleveland clinic cohort.

Fifty-four patients with localized prostate cancer, from 88 eligible participants, were prospectively enrolled in a neoadjuvant clinical trial (ClinicalTrials.gov NCT02770391) at the Cleveland Clinic (November 2016–April 2020; under an IRB-approved protocol, case 5815). Patients were genotyped for the *HSD3B1* (1245A/C) variant to determine adrenal-permissive (1245C) or adrenal-restrictive (1245A) alleles and were treated with apalutamide (240 mg daily) plus ADT. Peripheral blood was collected at baseline and 28 days posttreatment in 46 of the 54 enrolled patients, following standardized Cleveland Clinic protocols (22 1245A/A, 18 1245A/C, and 6 1245C/C); 8 patients were lost to follow-up. Untargeted metabolomic profiling (*n* = 18; 6 per genotype) was performed by Metabolon Inc. using UHPLC-MS. Subsequently, targeted liquid chromatography–tandem mass spectrometry (LC-MS/MS) quantification of 30 bile acids was performed in-house on serum samples from the entire cohort (*n* = 46), as previously described (67).

#### Dana-Farber cohort.

Serum samples were obtained from patients with prostate cancer enrolled at the Dana-Farber Cancer Institute prior to treatment and after 6 months of androgen deprivation and androgen pathway inhibitor therapy (ClinicalTrials.gov NCT02903368). The cohort included 35 *HSD3B1* (1245A/A) and 8 *HSD3B1* (1245 C/C) individuals. Circulating bile acids, including CA and GCDCA, were detected using LC-MS/MS following our previously validated steroid measurement protocol (68) adapted for bile acid detection.

### Cell culture

All cell lines were authenticated by short tandem repeat profiling and confirmed to be mycoplasma-free using the MycoStrip 100 assay (InvivoGen). Caco-2 cells (ATCC HTB-37) were maintained in Eagle’s minimum essential medium (EMEM) supplemented with 20% FBS. For differentiation, Caco-2 cells were switched to DMEM (4,500 mg/L d-glucose) supplemented with 20% FCS, 1% nonessential amino acids, 2 mM l-glutamine, and 100 U/mL penicillin with 100 μg/mL streptomycin. Cells were seeded onto 12 mm Transwell polyester membrane inserts (0.4 μm pore size; Corning, 3460) at 3–5 × 10^4^ cells per insert and maintained for 25–28 days (or 15–18 days after confluence for selected experiments). Monolayer integrity was confirmed by paracellular flux of lucifer yellow (Thermo Fisher Scientific, L453) (<1% after 5 hours).

HuH-7 cells (JCRB0403; RRID: CVCL_0336; obtained from the Japanese Collection of Research Bioresources Cell Bank, Osaka, Japan) and HEK293T cells (ATCC CRL-1573) were maintained in DMEM containing 4,500 mg/L d-glucose, supplemented with 10% FBS and 100 U/mL penicillin with 100 μg/mL streptomycin. HepG2 cells (ATCC HB-8065) were cultured in EMEM supplemented with the same additives. C4-2 cells (ATCC CRL-3314) were maintained in RPMI 1640 medium supplemented with10% FBS, 100 U/mL penicillin, and 100 μg/mL streptomycin. All cell lines were maintained at 37°C in a humidified 5% CO_2_ atmosphere.

### HIO preparation and culture

Primary intestinal stem cells were isolated from the lamina propria of histologically normal human ileum specimens, as previously described (69), and expanded in 3D culture according to a previous protocol (70), with minor modifications. Briefly, conditioned medium was prepared using the L-WRN supportive cell line (ATCC CRL-3276), which secretes Wnt3a, R-spondin 3, and Noggin, essential growth factors for maintaining intestinal epithelial stem cells. Early organoid establishment was supported with L-WRN–conditioned medium supplemented with Y-27632 (R&D Systems, 1254) and A 83-01 (Tocris Bioscience, 2939) for 2–3 days (70, 71), followed by differentiation in inhibitor-free L-WRN medium for 4–5 days. Mature organoids were treated with 100 nM DHEA for 24 or 48 h. Organoid and supernatants were collected for RT-qPCR, Western blot, and 3βHSD1 activity assays.

### Gene expression and transcript analysis

Total RNA was extracted using the RNeasy Mini Kit (Qiagen), and 800 ng was reverse transcribed with the iScript cDNA Synthesis Kit (Bio-Rad, 1708891). RT-qPCR was performed with iTaq Universal SYBR Green Supermix (Bio-Rad, 1725124) and 0.25 μM of each primer in 20 μL reaction on a QuantStudio 6 Pro Real-Time PCR system (Thermo Fisher Scientific). Cycling conditions were 95°C for 2 min, followed by 40 cycles of 95°C for 30 s, 60°C for 30 s, and 72°C for 30 s. Amplicon specificity was confirmed by melting curve analysis (60°C–95°C, 0.1°C/s ramp rate). Cq values were analyzed using QuantStudio Design and analysis software (v2.8.0; Thermo Fisher Scientific) and the 2^–ΔΔCt^ method, normalized to *RIBOSOMAL PROTEIN LATERAL STALK SUBUNIT P0* and controls. For TaqMan-based RT-qPCR, gene-specific primers and probes were synthesized by Integrated DNA Technologies. RT-qPCR was performed using PrimeTime Gene Expression Master Mix (Integrated DNA Technologies, 1055772) according to the manufacturer’s instructions. All experiments included 3 biological replicates. Primer and probe sequences are listed in Supplemental Table 2.

### Protein extraction and Western blotting

Cells were lysed in RIPA buffer with protease and phosphatase inhibitors (Thermo Fisher Scientific) and centrifuged at 1,200*g* for 30 min at 4°C. Protein concentrations were measured using the BCA Protein Assay Kit (Pierce, Thermo Fisher Scientific, 23227). Samples (25–30 μg) were separated on 10%–12% SDS-PAGE gels and transferred to Immobilon-P Transfer Membrane (MilliporeSigma). Membranes were blocked with 5% nonfat dry milk (Lab Scientific bioKemix, MSPP-M0841) in TBST for 1 h at room temperature and incubated overnight at 4°C with primary antibodies (3βHSD1, SLC10A2, LRH-1, AR, GR, 11βHSD1, 11βHSD2, and β-actin; listed in Supplemental Table 3), followed by HRP-conjugated secondary antibodies for 1 h at room temperature. Protein bands were detected using SuperSignal West Pico PLUS Chemiluminescent Substrate (Thermo Fisher Scientific), with β-actin as a loading control.

### Multiplex immunofluorescence and IHC

Multiplex immunofluorescence was performed on FFPE sections using the Opal Polaris Detection system (Akoya Biosciences), incorporating Opal 570 and Opal 690 fluorophores in a sequential multiplex protocol on the Discovery ULTRA automated staining platform (Roche Diagnostics). Sections underwent deparaffinization through 3 heating cycles at 69°C for 8 min each, followed by washing with Ventana Discovery Wash solution (Roche Diagnostics). Antigen retrieval was performed by treating the sections with Tris/borate/EDTA buffer (Discovery CC1, Roche; pH 8.0–8.5) at 95°C for 32 min, followed by endogenous peroxidase blocking. Between each staining cycle, antigen stripping was carried out using a citrate-based buffer (Discovery CC2, Roche Diagnostics). Primary antibodies were anti-SLC10A2 (1:50 dilution, 1 h at room temperature) and anti-3βHSD1(1:250 dilution, 1 h at room temperature). Both antibodies underwent antigen retrieval using CC1 buffer at 95°C for 32 min. Signal amplification was performed using OmniMap anti-rabbit HRP (Roche Diagnostics, 05269679001) for SLC10A2 and OmniMap anti-mouse HRP (Roche Diagnostics, 05269652001) for 3βHSD1, in combination with Opal 570 and Opal 690 fluorophores (Akoya Biosciences), respectively. Following staining, slides were counterstained with Spectral DAPI (Akoya Biosciences, FP1490) and mounted with ProLong Gold Antifade Mountant (Molecular Probes, Thermo Fisher Scientific).

IHC was performed on the Leica Bond RXm automated research stainer (Leica Biosystems). FFPE sections were deparaffinized at 60°C using BOND Dewax Solution, followed by antigen retrieval with either BOND Epitope Retrieval Solution 1 (pH 6.0; 20 min) or Solution 2 (pH 9.0; 30 min). Primary antibodies against AR (Cell Marque, 200R-18, RTU), GR (Cell Signaling Technology; 1:200 dilution), and NR5A2 (LRH-1) (Proteintech; 1:400 dilution) were then applied. The BOND Polymer Refine HRP Plex Detection Kit (Leica CDS9914) and hematoxylin counterstaining were used for detection. Whole-slide images were acquired with the Leica Aperio AT2 System (Leica Biosystems) and visualized with Aperio ImageScope. All antibodies are listed in Supplemental Table 3.

### Confocal microscopy

Super-resolution imaging was performed on a Zeiss LSM980 inverted laser scanning confocal microscope (Zeiss) with an Airyscan 2 super-resolution detector, achieving approximately 120 nm lateral resolution. Images were acquired in super-resolution mode using a ×63 Plan-Apochromat oil-immersion objective (NA 1.4) with ×2.5 crop mode (an effective magnification of ×157.5). Fluorophores were excited at 405, 488, and 561 nm with 2x line averaging. Both Airyscan and image processing, including LUT and linear brightness/contrast adjustments, was performed using Zen Black Software (Zeiss).

### Generation of CRISPR/Cas9-edited Caco-2 cell lines

CRISPR/Cas9 constructs expressing gRNAs targeting *HSD3B1* (s*gRNA-HSD3B1*: 5′-CCTTTCTGCTAGTATAAACG-3′; VectorBuilder) and *NR5A2* (s*gRNA-NR5A2*: 5′-ATGCGATCGAGCCAGTCCCA-3′ and 5′-TGGGGAACAGGGCCAGATGC-3′; Applied Biological Materials, 32145111) were designed and used to generate CRISPR/Cas9-disrupted Caco-2 cells. Lentiviral particles were produced by cotransfecting HEK293T cells with CRISPR/Cas9 and packaging plasmids (pMD2.G and psPAX2) using FuGENE HD (Promega, E2311). Viral supernatants collected at 48 and 72 h after transfection were filtered (0.45 μm) and used to transduce Caco-2 cells in the presence of 8 μg/mL polybrene. Stable cell populations were selected with 7 μg/mL puromycin (InvivoGen, ant-pr-1) for 7 days. Knockout efficiency was verified by RT-qPCR using gene-specific primers (Supplemental Table 2) and by Western blotting. Functional loss of 3βHSD1activity was further confirmed by LC-MS–based steroid conversion assay.

*NR3C1* (GR) knockout Caco-2 cells were generated using pLentiCRISPRv2 following the Zhang Lab CRISPR cloning protocol (https://www.addgene.org/crispr/zhang/), with reagents and guidance from Mehdi Baratchian (Cleveland Clinic Foundation) and Jianneng Li (University of Notre Dame, Notre Dame, Indiana, USA). The *NR3C1*-targeting gRNA was 5′-ATGACAACTTGACTTCTCTG-3′, and the nontargeting control guide (5′-ATCTGCCATGGCGTCCTGGC-3′) was used in parallel.

### Quantification of androgens and estrogens

Media and cell pelleted samples were stored at –80°C until analysis. Samples were spiked with a panel of isotopically labeled internal standards — androstene-3,17-dione-2,3,4-^13^C_3_​, 5α-dihydrotestosterone-16,17,17-d3, testosterone-16,17,17-d3​, cortisol-9,11,12,12-d4​, and 17β-estradiol-2,3,4-^13^C_3_​ — and extracted via liquid–liquid extraction with methyl tert-butyl ether. The organic phase was collected, evaporated, and reconstituted in 50% methanol for LC-MS/MS analysis. To accommodate distinct ionization characteristics and chromatographic condition requirements, each sample was injected twice. Androgens and estrogens were analyzed following previously validated steroid measurement protocols (42, 72). Data were acquired and processed using Sciex OS (v3.3.1.43).

### Steroid metabolism assay and HPLC analysis

Differentiated Caco-2 cells or 4–5 × 10^4^ cells of other lines seeded on the poly-l-ornithine–coated 12-well plates (A-004-C, Sigma-Aldrich) were incubated with [³H]-DHEA (PerkinElmer, CUST86441000MC, 12 nM, 3–6 × 10^5^ cpm), [³H]-cortisol (revvity, NET396250U, 12 nM, 3–6 × 10^5^ cpm), and [³H]-cortisone (American Radiolabeled Chemicals, ART 0743-250uCi, 12 nM, 3–6 × 10^5^ cpm) at 37°C. Culture medium was collected at 24 or 48 h, and conjugated metabolites were hydrolyzed with β-glucuronidase (Sigma-Aldrich, G7646-500KU) for ≥2 h at 37°C. Steroids were extracted with ethylacetate/isooctane (1:1, v/v) or ethylacetate alone, dried, and reconstituted in 50% methanol for HPLC analysis. Metabolites were separated using an Agilent 1260 HPLC system (Agilent Technologies). Radioactivity was detected online with a β-RAM Model 5 detector (LabLogic Systems) and Liquiscint scintillation cocktail (National Diagnostics, LS12120LTR). Radiochromatographic data were analyzed using Laura software (LabLogic Systems).

### TCA transport assay

Differentiated Caco-2 monolayers were rinsed and preincubated with prewarmed HBSS (Sigma-Aldrich, 55037C) for 15 min at 37°C. Transport was initiated by adding HBSS containing [³H]-TCA (PerkinElmer) to the apical compartment, with HBSS in the basolateral chamber (well). After 3 or 5 h, basolateral aliquots were collected to quantify transcellular [³H]-TCA flux. For HPLC analysis, extracts were reconstituted in 70% methanol and analyzed on an Agilent 1260 HPLC system. Chromatographic separation was performed at 30°C using a methanol-water gradient. Radioactivity was detected online with a β-RAM Model 5 detector and Liquiscint scintillation cocktail. Radiochromatographic data were analyzed using Laura software.

### scRNA-seq and analysis

We reanalyzed our previously published scRNA-seq atlas (73). UMAP plots were generated using R version 4.4.1 (74) and R packages including Seurat v. 5.2.1 (75–79), tidyverse v. 2.0.0 (80), and patchwork v. 1.3.0 (81).

### Statistics

Data were analyzed using GraphPad Prism v10.5 (GraphPad Software). Comparisons between 2 groups were performed using 2-tailed Student’s *t* tests or Mann-Whitney *U* tests, as appropriate. For experiments involving more than 2 groups compared with a common control, statistical significance was determined using 1-way ANOVA followed by Dunnett’s multiple-comparison test to correct for multiple comparisons. *P* values are indicated as *P < 0.05, **P < 0.01, ***P < 0.001, and ****P < 0.0001. Data are presented as mean ± SD or median ± SD (as indicated), with each point representing an individual subject. Significant differences versus control are indicated by horizontal lines with asterisks.

### Study approval

The studies involving human participants were reviewed and approved by the IRB of the Cleveland Clinic (clinical protocol numbers CASE5815 and CASE7813). All participants provided written informed consent prior to enrollment in the study.

### Data availability

Values for all data points are reported in the Supporting Data Values file. The deidentified clinical data generated and analyzed during this study are available upon request from the corresponding author, subject to institutional approval and in accordance with regulations governing the use of human subject data. The single-cell transcriptomics dataset used in this study was generated by the Florian Rieder laboratory (73) from human patient samples and is subject to institutional restrictions imposed by the Cleveland Clinic. These data are not publicly deposited; however, they can be shared by the originating laboratory upon reasonable request and following approval through the Cleveland Clinic’s data access committee. Requests should be directed to Florian Rieder (riederf@ccf.org). Data sharing will be facilitated in accordance with the NIH Federal Demonstration Partnership Data Transfer and Use Agreement. Processed data sufficient to reproduce the analyses in this manuscript are available upon request from the corresponding author.

## Author contributions

NS and NF conceptualized and led the study and wrote the original paper. NF, MA, GW, YMC, PKM, RAB, and SJ developed the methodology and acquired data. NF and RD performed experiments, with MA and YMC conducting mass spectrometry and data analysis. NS and NF performed additional data analyses. AA conducted statistical analyses. NS provided resources for the project. FR, MET, and RRM provided human single-cell and Dana-Farber bile acid datasets, as well as critical feedback. All authors reviewed and approved the final submission.

## Conflict of interest

The authors have declared that no conflict of interest exists.

## Funding support

This work is the result of NIH funding, in whole or in part, and is subject to the NIH Public Access Policy. Through acceptance of this federal funding, the NIH has been given a right to make the work publicly available in PubMed Central.

- NIH grants R01CA261995 and R01CA172382 (to NS) and P30 DK097948 (National Institute of Diabetes and Digestive and Kidney Diseases) (to FR).
- Prostate Cancer Foundation.

## Supplementary Material

Supplemental data

Unedited blot and gel images

Supporting data values

## Version 1 06/15/2026

Electronic publication
