# Supplementary material for: HSD3B1 links ileal steroid metabolism to bile acid regulation in patients with prostate cancer
Source: J Clin Invest. 2026 Jun 15;136(12):e202725. doi: 10.1172/JCI202725 (PMC13262715; doi:10.1172/JCI202725)
Supplement: Supplemental data [file jci-136-202725-s007.pdf]

Supplemental Data

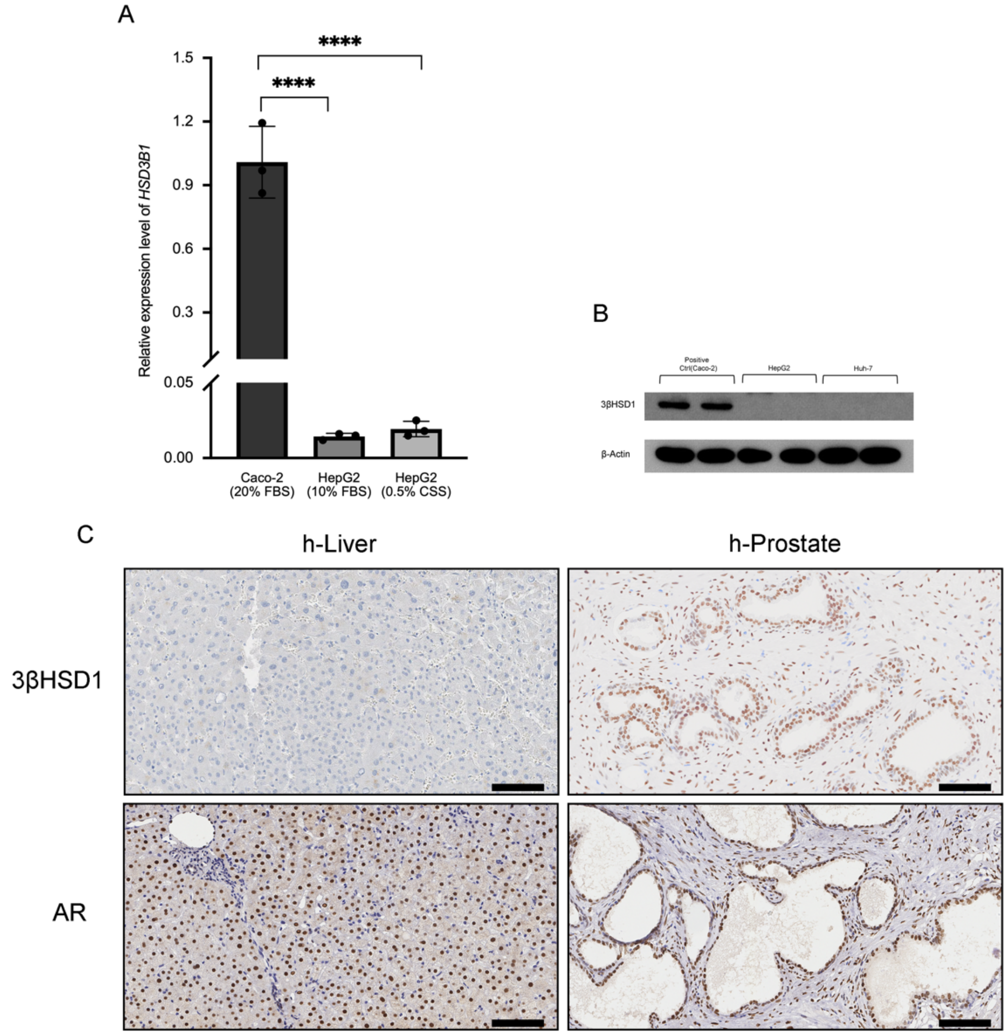

Supplemental Figure 1. Absence of *HSD3B1* transcript and 3βHSD1 protein in hepatic carcinoma cells and human hepatocytes. (**A**) qRT-PCR analysis shows negligible *HSD3B1* mRNA expression in HepG2 cells. (**B**) western blot analysis confirms absence of detectable 3βHSD1 protein in both HepG2 and HuH-7 cell lysates. (**C**) Immunohistochemical staining of human liver tissue demonstrates absence of 3βHSD1 and presence of androgen receptor (AR) (h-prostate: positive control). (**C**) Scale bare: 100 μm. Data are presented as mean ± SD; n = 3 independent experiments. Statistical significance was determined using one-way ANOVA followed by Dunnett's multiple-comparisons test; ****p < 0.0001. The human liver image shown in the top left panel is the same sample previously presented in Figure 2B and is reproduced here for comparison.

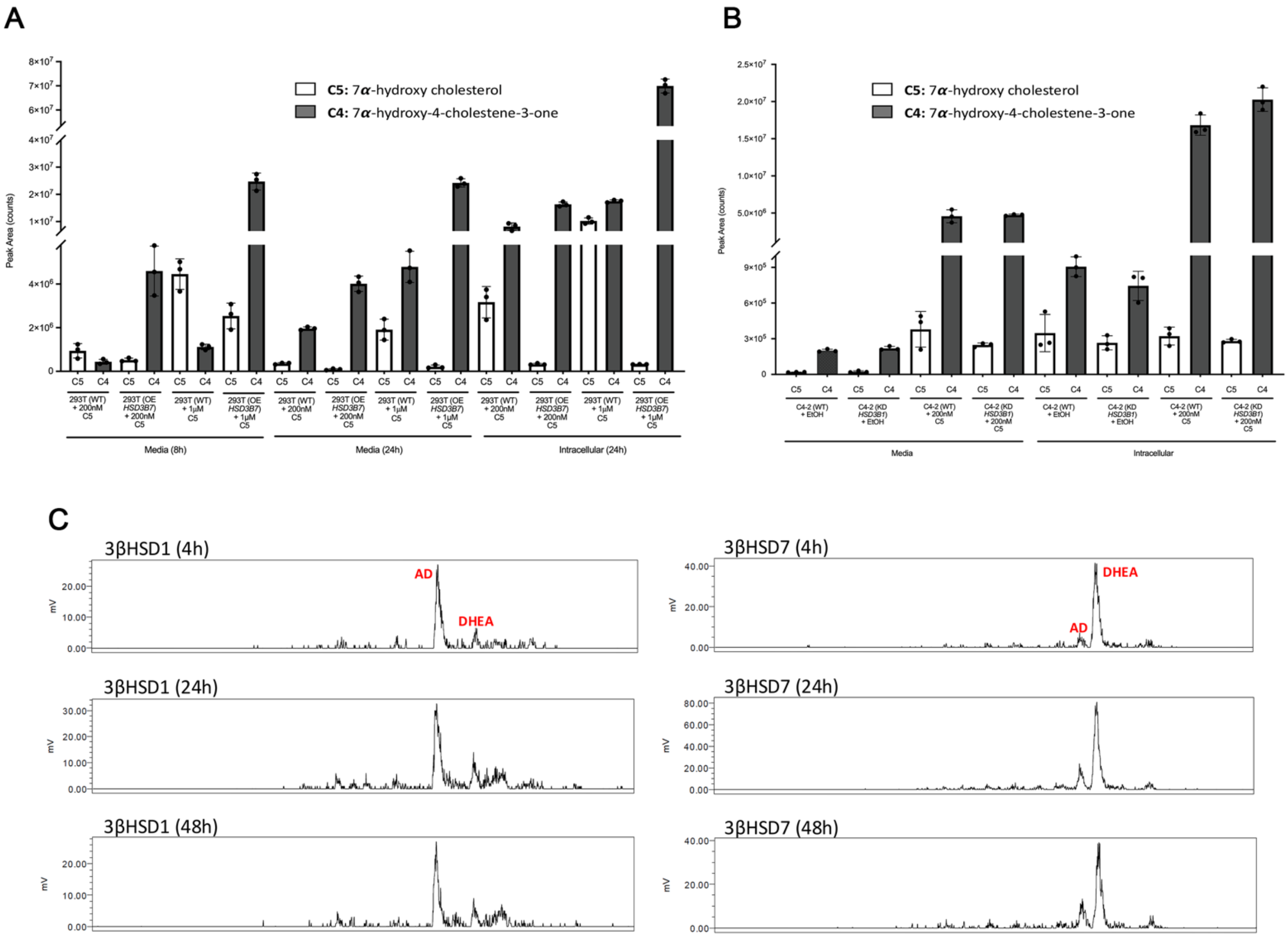

Supplemental Figure 2. Functional dissociation between 3βHSD1 & 3βHSD7 activities. **(A)** HEK293T cells overexpressing *HSD3B7* converted C5 to C4 actively in a substrate- and time-dependent manner. **(B)** *HSD3B1* knockdown in C4-2 cells, compared with parental C4-2 cells, did not alter C4 synthesis after C5 treatment, suggesting 3βHSD7 activity is 3βHSD1-independent. (**C**) HEK293T cells expressing 3βHSD7 or 3βHSD1 were treated with dehydroepiandrosterone (DHEA); only 3βHSD1 metabolized DHEA to androstenedione (AD) and testosterone (T). Data are presented as mean ± SD (n = 3).

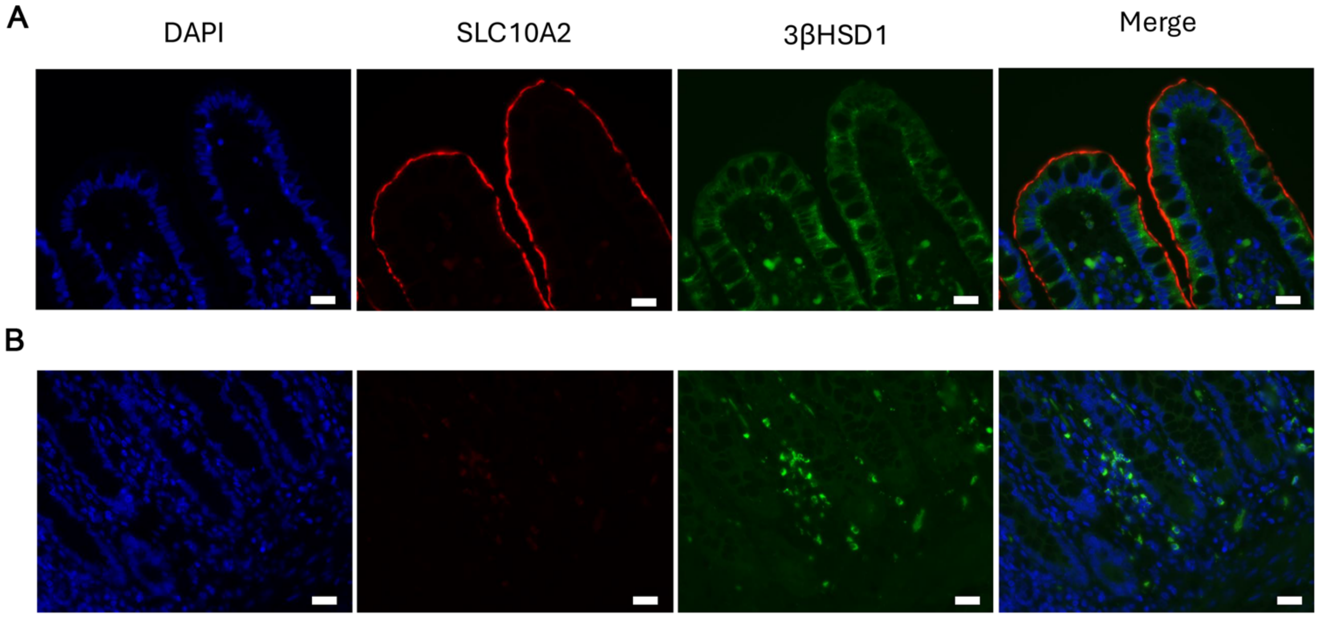

Supplemental Figure 3. 3βHSD1 and SLC10A2 are expressed in human terminal ileum. Immunofluorescence staining of human terminal ileum shows 3βHSD1 (green) and SLC10A2 (red) localization in villus tips. (**A**=villus tip and **B**= crypts) Nuclei were counterstained with DAPI (blue). Representative images from (n=5) independent samples are shown. Scale bar: 20 μm.

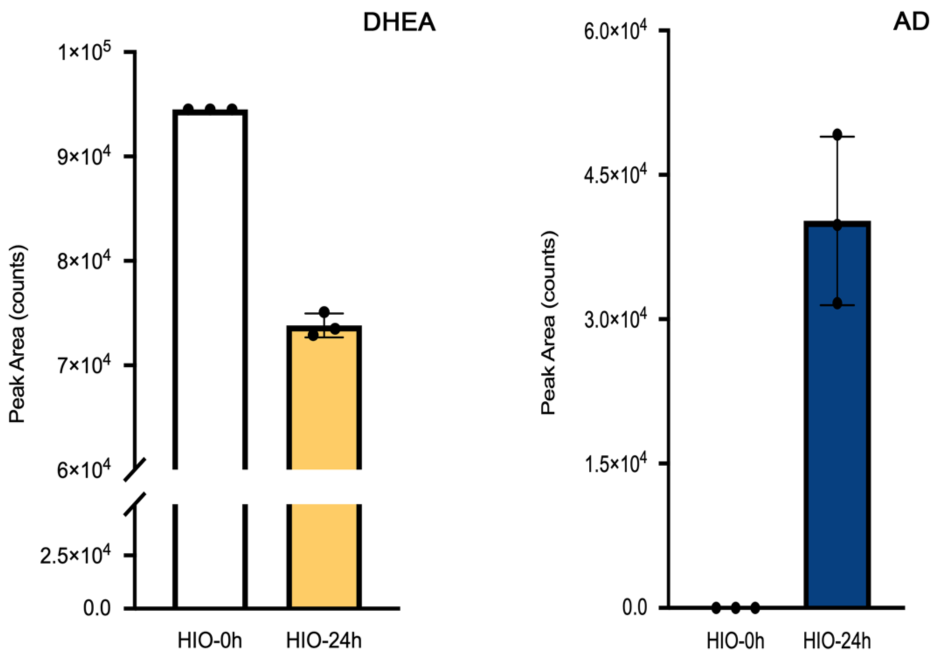

Supplemental Figure 4. 3βHSD1 activity in human intestinal organoids (HIOs). Human ileal organoids treated with DHEA (100 nM) for 24 h showed robust formation of androstenedione (AD), as measured by mass spectrometry, indicating active 3βHSD1-mediated steroidogenesis. Data are presented as mean ± SD; n=3 independent experiments.

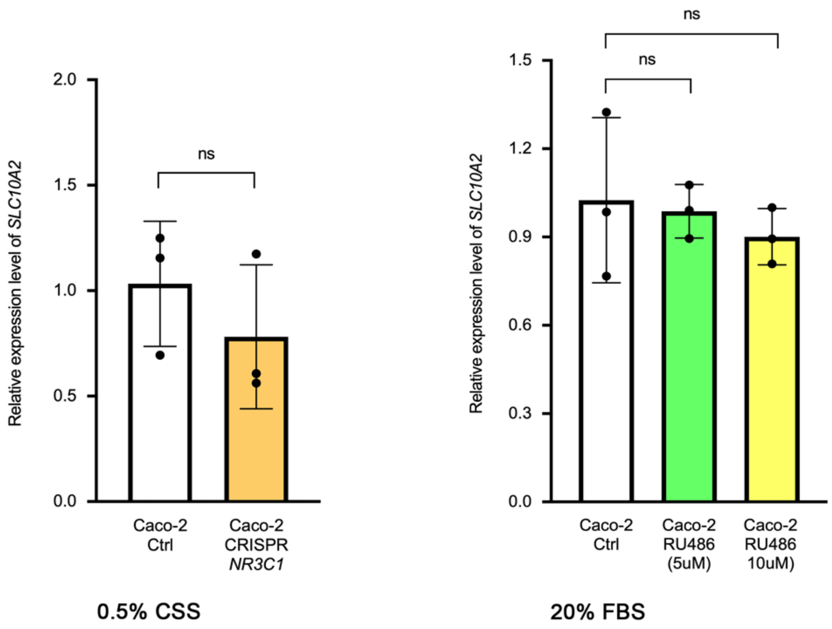

Supplemental Figure 5. ***SLC10A2*** **remains LRH-1-reposnsive despite glucocorticoid receptor (GR) inhibition.** In Caco-2 cells under charcoal-stripped conditions, *SLC10A2* expression was sustained by LRH-1 activation even with GR blockade. Data are presented as mean ± SD; n=3 independent experiments. Statistical significance was determined using a two-tailed Student's *t*-test or, for comparisons of more than two groups with a common control, one-way ANOVA followed by Dunnett's multiple-comparisons test; ns: non-significant.

Supplemental Table

Bile acid (nM)	Type	Baseline Median	Baseline Mean	Post-treatment Median	Post-treatment Mean
Glycochenodeoxycholic Acid	Primary (conj.)	757.50	1008.00	310.10	401.00
Deoxycholic Acid	Secondary	397.80	660.50	267.40	392.80
Chenodeoxycholic Acid	Primary	383.70	648.20	267.50	389.40
ω-Muricholic Acid	Primary	321.40	640.70	303.60	502.10
Glycodeoxycholic Acid	Secondary (conj.)	319.20	475.90	153.40	251.40
Ursodeoxycholic Acid	secondary	167.60	411.20	50.28	117.40
Glycocholic Acid	Primary (conj.)	164.70	278.50	120.10	169.10
Hyodeoxycholic Acid	Secondary	112.60	263.00	118.90	195.80
Taurochenodeoxycholic Acid	Primary (conj.)	69.73	125.10	58.41	93.04
Glycoursodeoxycholic Acid	Secondary (conj.)	66.78	157.40	20.29	45.86
Hyocholic Acid	Primary	52.74	272.00	90.72	360.20
Cholic Acid	Primary	42.42	294.60	29.67	219.90
Taurodeoxycholic Acid	Secondary (conj.)	32.86	67.28	39.29	67.92
Isolithocholic Acid	secondary	30.85	44.81	12.24	17.62
7-Ketolithocholic Acid	secondary	30.20	47.16	31.44	39.30
Lithocholic Acid	Secondary	24.43	35.71	16.38	22.15
Taurocholic Acid	Primary (conj.)	22.29	50.21	22.57	69.43
3-Ketodeoxycholic Acid	Secondary	18.20	24.07	13.01	20.48
Glycolithocholic Acid	Secondary (conj.)	14.46	27.73	4.74	7.68
Glycohyocholic Acid	Primary (conj.)	9.07	11.25	21.21	37.29
A-Muricholic Acid	Primary	6.07	29.37	15.59	39.20
Taurolithocholic Acid	Secondary (conj.)	4.57	6.06	3.80	4.05
Glycohyodeoxycholic Acid	Secondary (conj.)	2.52	4.68	2.63	3.65
7-Ketodeoxycholic Acid	secondary	2.18	9.11	1.83	4.99
Tauroursodeoxycholic Acid	Secondary (conj.)	2.15	4.86	1.44	2.96
Tauro-ω-Muricholic Acid	Primary (conj.)	1.95	5.47	6.13	13.22
Tauro-α-Muricholic Acid	Primary (conj.)	1.69	4.38	8.58	16.34
Tauro-β-Muricholic Acid	Primary (conj.)	1.63	4.43	13.00	22.83
β-Muricholic Acid	Primary	0.84	21.78	1.81	24.16
Taurohyodeoxycholic Acid	Secondary	0.26	1.91	0.26	1.91

Supplemental Table 1. **Serum bile acid profiles in prostate cancer patients before and after ADT plus apalutamide.** Primary and secondary bile acids including conjugated forms, were quantified at baseline and after 28 days of treatment. Values are presented as median and mean concentrations (nM; n=46 patients).

Gene (Protein)	Forward primer	Reverse Primer
*HSD3B1*TaqMan (3βHSD1)	CACACAGCAAAAAGCTTGCTGAG	GTTGTTCAGGGCCTCGTTTATACTAG
HSD3B1 TaqMan Probe	56-FAM/TAAGGCACA/ZEN/AGTGTACAGGGTGCCGCC/3IKBkFQ	
RPLP0 (RPLP0)	ATTACACCTTCCCACTTGCTG	ACTCTTCCTTGGCTTCAACCTTA
RPLP0 TaqMan Probe	56-FAM/AGGCCTTCT/ZEN/AGGCCTTCTTGGCTGATCCATCTGC/3IKBkFQ	
HSD3B1 (3βHSD1)	AGAAGAGCCTCTGGAAAACACATG	TAAGGCACAAGTGTACAGGGTGC
HSD3B2 (3βHSD1)	AGAAGAGCCTCTGGAAAACACATG	CGCACAAGTGTACAAGGTATCACCA
SLC10A2 (SLC10A2)	CTGGTTTCTCTCGTTGTTCCTG	CCTCCAACCACAGCTATGAGC
NR5A2 (LRH-1)	CACTCTGCCTCCAAAGGCCT	GGAAAGTGGCCATATGTTTGGTAACCT
NR3C1 (GR)	CTAATGGCTATTCAAGCCCCAGCAT	GTGCTGTCCTTCCACTGCTCT
HSD11B1 (11βHSD1)	GAGGTTCTCTCTGTGTGTCCT	GTAGTAGGCCATGAAGAGCCC
HSD11B2 (11βHSD2)	TGGATCGCGTTGTCCCG	GTTCAACTCCAATACGGTGGC

Supplemental Table 2. qPCR primers and probes used in this study.

Antibodies		Info
3βHSD1	Primary	ab55268 [3C11-D4], Abcam, Waltham, MA
SLC10A2 (WB)	Primary	25245-1-AP, Proteintech, Rosemont, IL
SLC10A2 (IHC/IF)	Primary	HPA004795, Sigma-Aldrich, St. Louis, MO
AR (WB)	Primary	5153S (D6F11), Cell Signaling Technology, Danvers, MA
AR (IHC)	Primary	200R-18 (SP107), Cell Marque, Rocklin, CA
GR	Primary	12041S (D6H2L), Cell Signaling Technology, Danvers, MA
LRH-1	Primary	22460-1-AP, Proteintech, Rosemont, IL
11βHSD1	Primary	ab39364, Abcam, Waltham, MA
11βHSD2	Primary	sc-365529 (C9), Santa Cruz Biotechnology, Dallas, TX
β-Actin	Primary	4970S (13E5), Cell Signaling Technology, Danvers, MA 3700S (8H10D10), Cell Signaling Technology, Danvers, MA
HRP-conjugated anti-Mouse	Secondary	NA931, Cytiva, Marlborough, MA
HRP-conjugated anti-Rabbit	Secondary	31460, Thermo Fisher Scientific, Waltham, MA
OmniMap anti-Rabbit HRP	Secondary	05269679001, Roche, Indianapolis, IN
OmniMap anti-Mouse HRP	Secondary	05269652001, Roche, Indianapolis, IN
Opal 570 & Opal 690 (TSA)	Fluorophore	Akoya Biosciences, Menlo Park, CA

Supplemental Table 3. Primary and secondary antibodies used in this study.
