# Supplementary material for: HSD3B1 links ileal steroid metabolism to bile acid regulation in patients with prostate cancer
Source: J Clin Invest. 2026 Jun 15;136(12):e202725. doi: 10.1172/JCI202725 (PMC13262715; doi:10.1172/JCI202725)
Supplement: Unedited blot and gel images [file jci-136-202725-s008.pdf]

Unedited blots

Please note that the original full blots have been cut in thin pieces before the incubation with the appropriate antibody.

The pictures represent the unmodified images as originally acquired

(some blots scanned in black-&-white and others in color printer).

Full unedited blot for **Figure 2F**

3βHSD1 **~42 kDa**

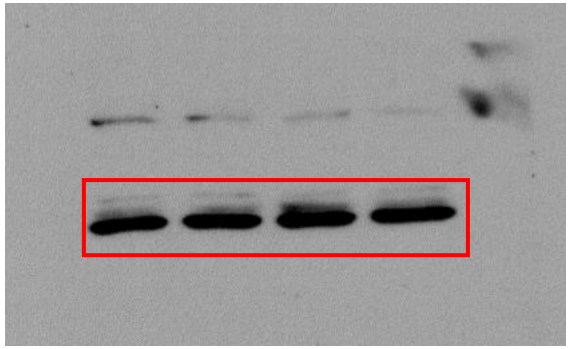

β-Actin **~45 kDa**

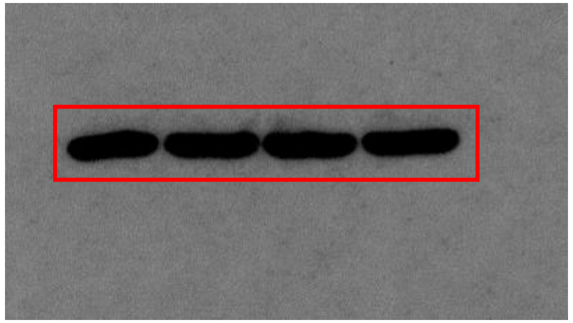

SLC10A2 **~38 kDa**

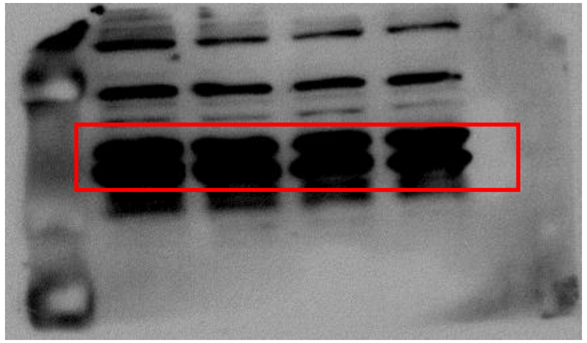

β-Actin **~45 kDa**

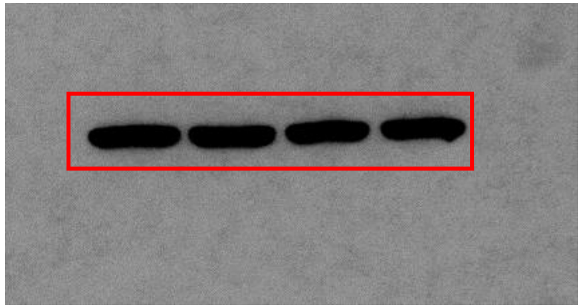

All four lanes: differentiated Caco-2

Full unedited blot for **Figure 3E**

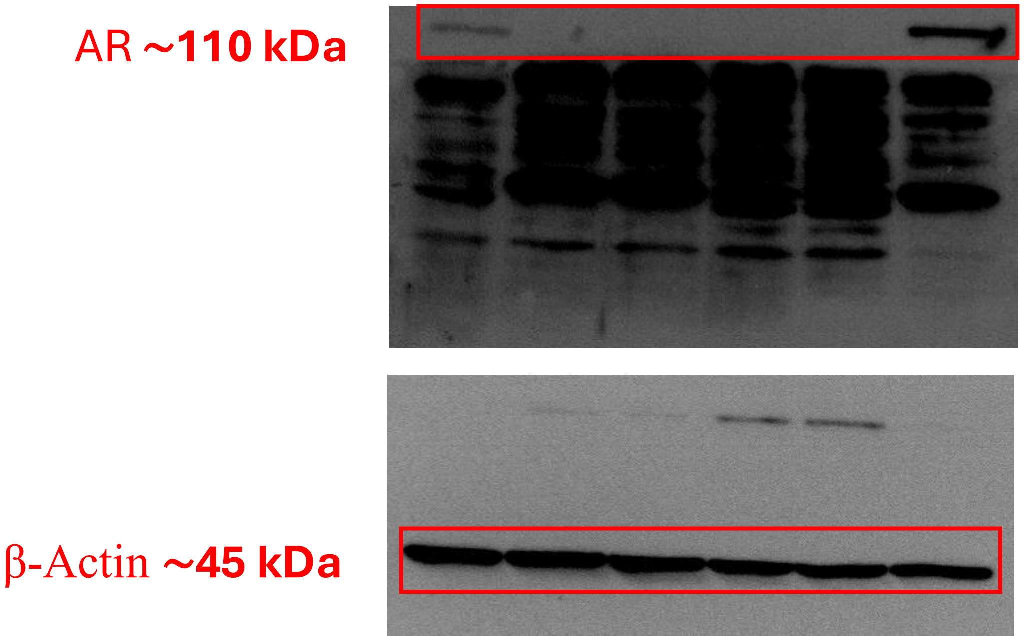

Positive Ctrl, undifferentiated Caco2 (2x), differentiate Caco-2 (2x), Positive Ctrl

Full unedited blot for **Figure 4B**

LRH-1 **~68 kDa**

β-Actin **~45 kDa**

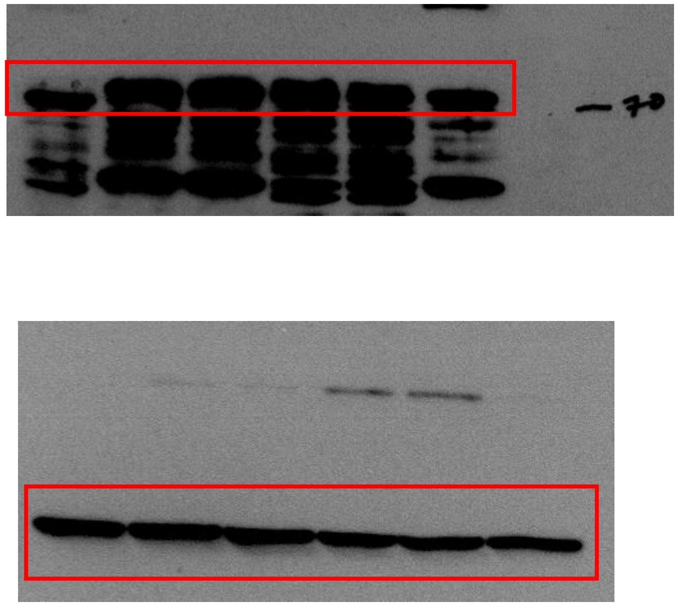

Positive Ctrl, undifferentiated Caco2 (2x), differentiate Caco-2 (2x), Positive Ctrl,

Full unedited blot for **Figure 5B**

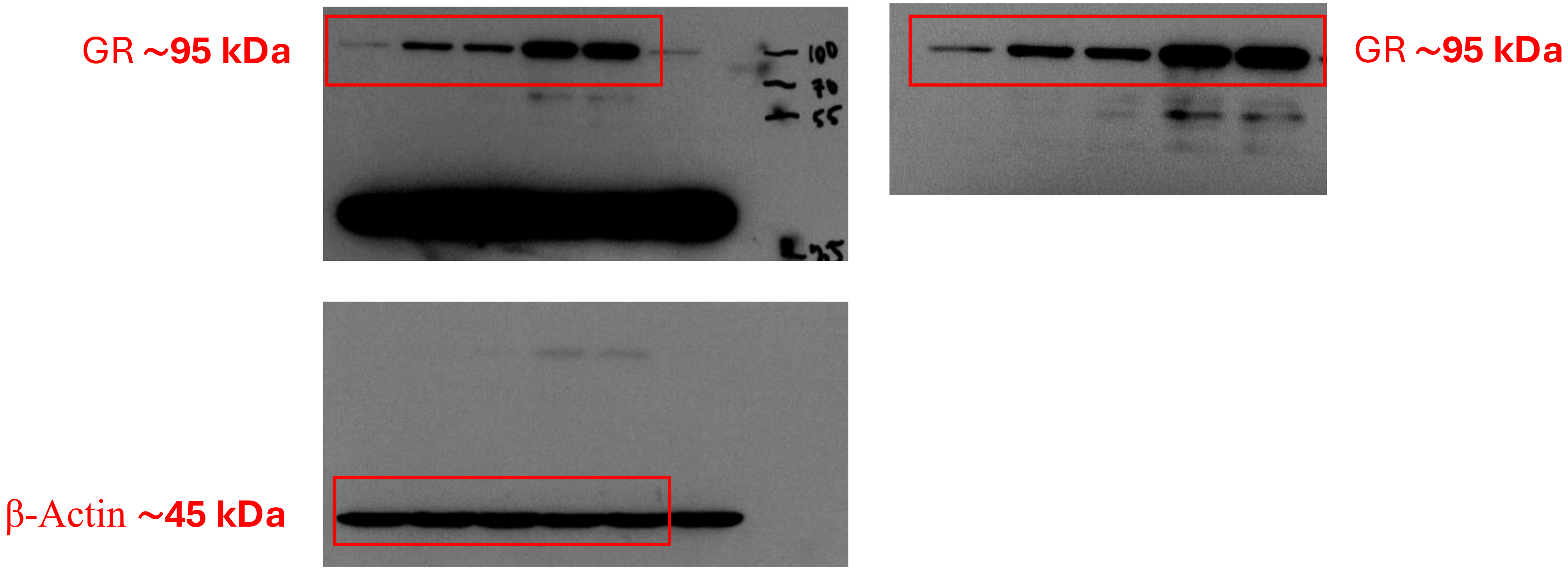

Positive Ctrl, undifferentiated Caco2 (2x), differentiate Caco-2 (2x)

Full unedited blot for **Figure 5C**

GR **~95 kDa**

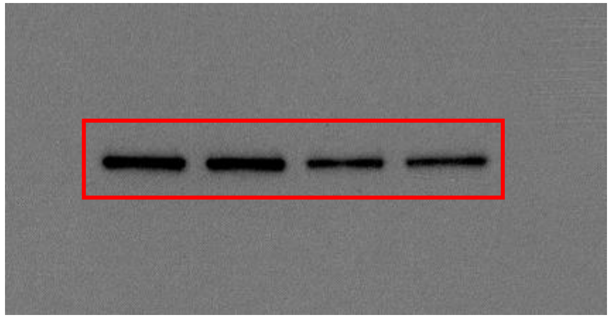

β-Actin **~45 kDa**

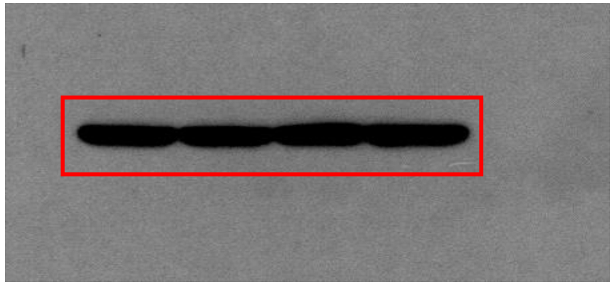

Caco-2 Ctrl (2x), Caco-2 + ML-180 (2x)

Full unedited blot for **Figure 5D**

SLC10A2 **~38 kDa**

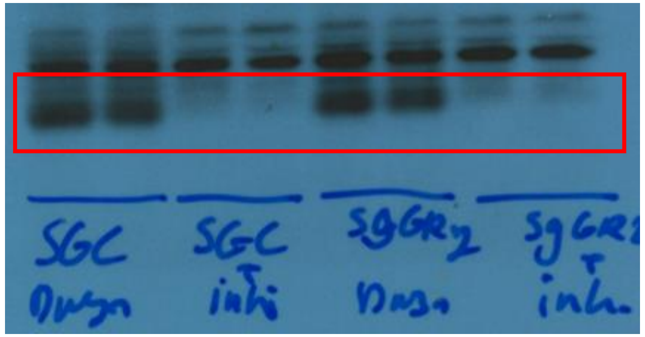

β-Actin **~45 kDa**

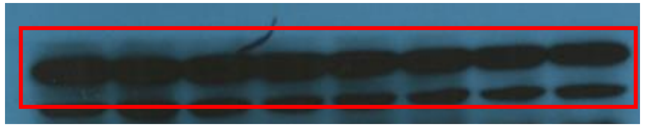

Caco-2 Ctrl (DMSO), Caco-2 Ctrl (ML-180), Caco-2 GR KD (DMSO), Caco-2 GR KD (ML-180),

Full unedited blot for **Figure 5E**

11βHSD2 **~42 kDa**

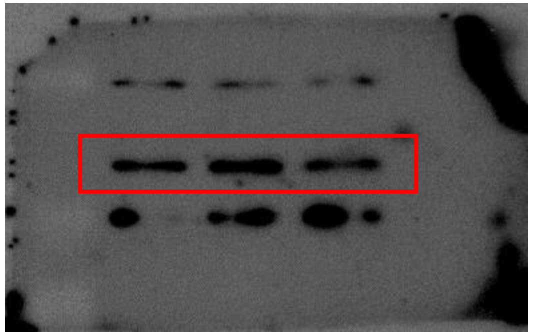

β-Actin **~45 kDa**

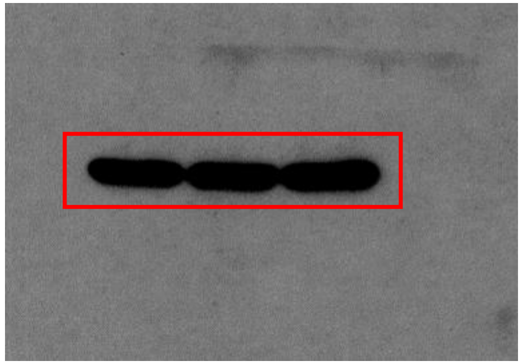

11βHSD1 **~38 kDa**

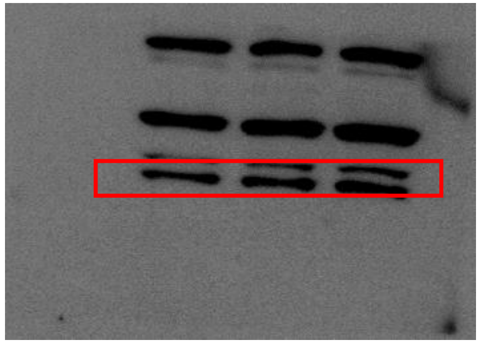

β-Actin **~45 kDa**

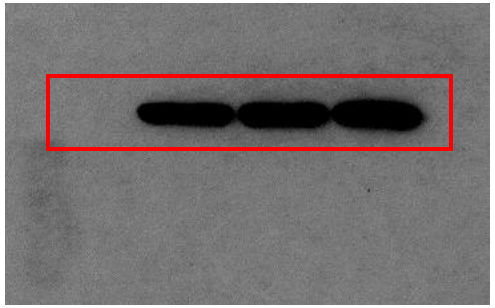

Differentiated Caco-2 (3x)

Full unedited blot for **Figure 5F**

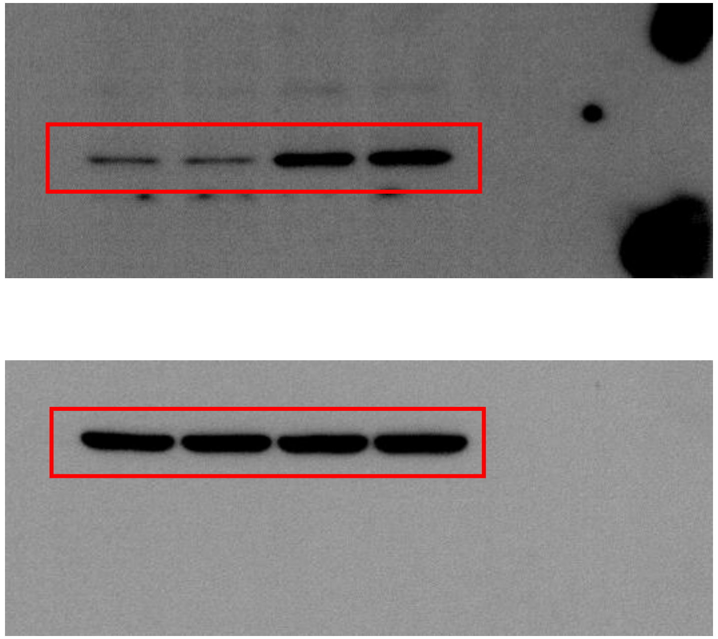

Caco-2 Ctrl (2x), Caco-2 + ML-180 (2x)

Full unedited blot for **Figure Supplemental Figure 1B**

3βHSD1 **~42 kDa**

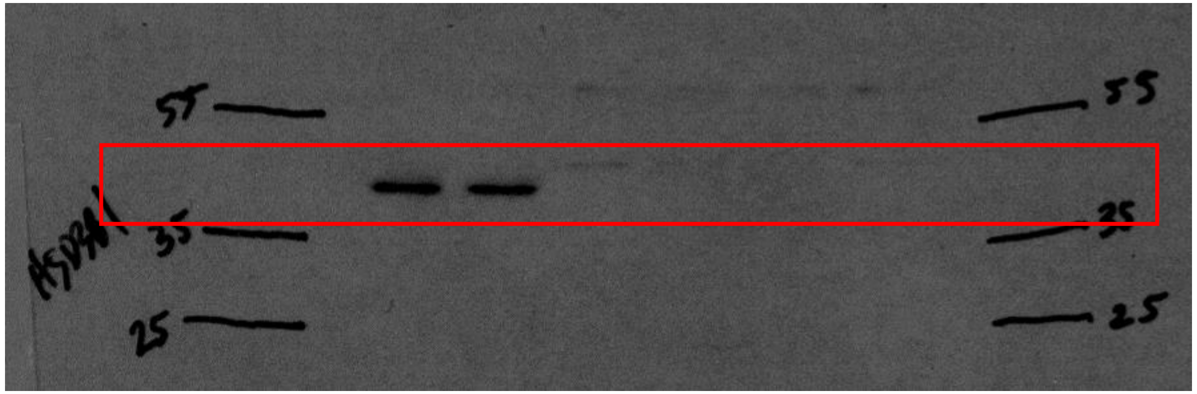

β-Actin **~45 kDa**

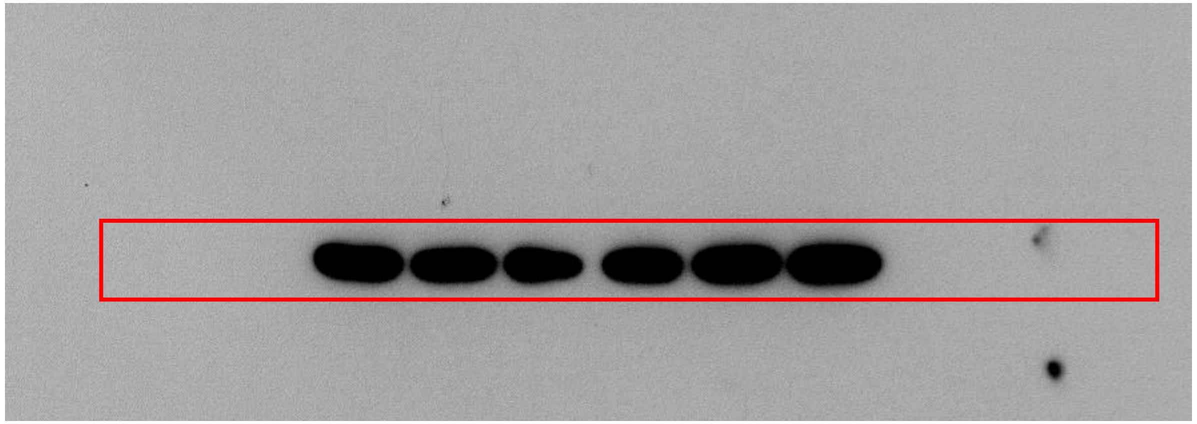

Positive Ctrl (2x), HepG2 (2x), Huh-7 (2x)
